# Temporal Analysis of Gene Expression in the Murine Schwann Cell Lineage and the Acutely Injured Postnatal Nerve

**DOI:** 10.1371/journal.pone.0153256

**Published:** 2016-04-08

**Authors:** Anjali Balakrishnan, Morgan G. Stykel, Yacine Touahri, Jo Anne Stratton, Jeff Biernaskie, Carol Schuurmans

**Affiliations:** 1 Department of Biochemistry and Molecular Biology, Cumming School of Medicine, Alberta Children’s Hospital Research Institute and Hotchkiss Brain Institute, University of Calgary, Calgary, AB, T2N 4N1, Canada; 2 Department of Comparative Biology and Experimental Medicine, Faculty of Veterinary Medicine, Alberta Children’s Hospital Research Institute and Hotchkiss Brain Institute, University of Calgary, Calgary, AB, T2N 4N1, Canada; 3 Department of Clinical Neurosciences, Cumming School of Medicine, Alberta Children’s Hospital Research Institute and Hotchkiss Brain Institute, University of Calgary, Calgary, AB, T2N 4N1, Canada; Centro Cardiologico Monzino, ITALY

## Abstract

Schwann cells (SCs) arise from neural crest cells (NCCs) that first give rise to SC precursors (SCPs), followed by immature SCs, pro-myelinating SCs, and finally, non-myelinating or myelinating SCs. After nerve injury, mature SCs ‘de-differentiate’, downregulating their myelination program while transiently re-activating early glial lineage genes. To better understand molecular parallels between developing and de-differentiated SCs, we characterized the expression profiles of a panel of 12 transcription factors from the onset of NCC migration through postnatal stages, as well as after acute nerve injury. Using Sox10 as a pan-glial marker in co-expression studies, the earliest transcription factors expressed in E9.0 Sox10^+^ NCCs were Sox9, Pax3, AP2α and Nfatc4. E10.5 Sox10^+^ NCCs coalescing in the dorsal root ganglia differed slightly, expressing Sox9, Pax3, AP2α and Etv5. E12.5 SCPs continued to express Sox10, Sox9, AP2α and Pax3, as well as initiating Sox2 and Egr1 expression. E14.5 immature SCs were similar to SCPs, except that they lost Pax3 expression. By E18.5, AP2α, Sox2 and Egr1 expression was turned off in the nerve, while Jun, Oct6 and Yy1 expression was initiated in pro-myelinating Sox9^+^/Sox10^+^ SCs. Early postnatal and adult SCs continued to express Sox9, Jun, Oct6 and Yy1 and initiated Nfatc4 and Egr2 expression. Notably, at all stages, expression of each marker was observed only in a subset of Sox10^+^ SCs, highlighting the heterogeneity of the SC pool. Following acute nerve injury, Egr1, Jun, Oct6, and Sox2 expression was upregulated, Egr2 expression was downregulated, while Sox9, Yy1, and Nfatc4 expression was maintained at similar frequencies. Notably, de-differentiated SCs in the injured nerve did not display a transcription factor profile corresponding to a specific stage in the SC lineage. Taken together, we demonstrate that uninjured and injured SCs are heterogeneous and distinct from one another, and de-differentiation recapitulates transcriptional aspects of several different embryonic stages.

## Introduction

The two main glial cell types in the peripheral nervous system (PNS) are Schwann cells (SCs) and satellite glial cells. During development, SCs wrap both myelinated and non-myelinated axons, with SCs coupled with axons greater than 1μm in diameter differentiating into myelinating SCs, while SCs connected to smaller diameter axons (<1μm) become non-myelinating Remak cells. Remak cells can enclose several axons, while myelinating SCs surround and myelinate only a single axonal length. SCs play an essential role in peripheral nerve regeneration and restoration of nervous function post-injury [1]. One of the most remarkable features of adult SCs is their capacity to transiently revert to an undifferentiated and proliferative state following nerve injury. In doing so, ‘de-differentiated’ SCs are able to assume a variety of critical roles to support nerve repair that include modulating the immune response [2, 3], secretion of trophic molecules [4–8], constructing growth permissive pathways for regenerating axons, and re-myelination of regenerating axons [9–11]. Previous work has collectively suggested that this de-differentiation process recapitulates SC development, such that ‘repair’ SCs resemble an embryonic immature Schwann cell (iSC) phenotype [10, 12]. However, the transcription factors that regulate this reversion to a reparative SC state are only partially understood.

SC development has been studied extensively, leading to the identification of distinct developmental stages. SCs originate from a multipotent, migratory population of neural crest cells (NCC) progenitor cells [13]. In mouse, NCCs emerge from the dorsal neural tube between embryonic day (E) 9.0 until E10.5, with trunk NCCs migrating along either dorso-lateral or ventral pathways. NCCs in the ventral pathway either migrate between the somites to reach the dorsal aorta, where they give rise to sympathetic chain ganglia and the aortic plexus, or migrate through the rostral somite to give rise to the dorsal root ganglia (DRG), adrenal medulla, and SCs [14]. NCCs also give rise to a subset of multipotent boundary cap cells that line the dorsal root entry zone and motor exit points, and which differentiate into SCs that populate the dorsal and ventral roots [15].

At approximately E12.5, migratory NCCs fated for a glial lineage give rise to SC precursors (SCPs) that populate the dorsal and ventral roots, DRG, and developing nerves [16]. SCPs are distinguished from migrating NCCs as they begin to express glial lineage markers, and they associate directly with growing axon bundles [10, 17]. As development proceeds, SCPs either give rise to immature SCs (iSCs), or alternately, endoneurial fibroblasts and melanocytes [18, 19]. iSCs first appear from E14.5 and persist until just before birth [10]. iSCs cluster to envelop and deposit basal lamina around developing axons. Cytoplasmic processes of the iSCs penetrate axonal bundles, dispersing larger axons to the outside of the bundle. Ultimately, iSCs in contact with large diameter axons will associate with a single axon, resulting in radial sorting [20]. Post radial sorting, iSCs in association with smaller diameter axon bundles become non-myelinating SCs [21]. iSCs associated with large diameter axons are a transient population termed pro-myelinating SCs; these are the SCs that will progress towards the myelinating stage. Pro-myelinating SCs generally appear just prior to birth, and rapidly increase in number by postnatal day (P) 1 in mice [16].

Several transcription factors have been implicated in regulating the progression from NCC to a mature SC. Importantly, the transcription factors known to be upregulated after injury are presumed to be those expressed at the SCP or iSC stage [10, 12]. However, a comprehensive analysis of the expression profiles of these transcription factors has not yet been conducted. Several of the markers have not been analysed through development or post-injury, while the expression profiles of other markers have not been studied in an *in vivo* setting. Instead, parallels between developmental and de-differentiated SC factors are often identified across various injury models in an assortment of species, timeframes, ages, each employing different processing techniques and reagents. Additionally, many of these comparisons are made at the level of mRNA, or *in-vitro*, making it difficult to accurately appreciate the *in vivo* de-differentiated phenotype, and how closely it recapitulates developmental SC programs. Here, we have described the transcriptional profile of developing and de-differentiated SC *in vivo* in a comparable and relevant manner. We conducted an extensive spatio-temporal analysis through five key stages of embryonic mouse development (E9.0, E10.5, E12.5, E14.5, E18.5), postnatal stages P7 and P65, as well as within the P65 nerve following acute injury, when SCs are actively acquiring a reparative state.

To identify expression patterns characteristic of each developmental stage, we examined a panel of 12 transcription factors previously implicated in NCC or SC development, including Sox2 [22–24], Sox9 [25–27] and Sox10 [26, 28–34], members of the SRY (sex determining region Y)-box (Sox) family of HMG-box transcription factors, Egr1/Krox24 and Egr2/Krox20 [15, 35], EGR class zinc finger proteins, Yy1 (Yin Yang 1) [36], a Gli-Kruppel zinc finger protein, Oct6/Pou3f1 [16, 37, 38], a POU domain class 3 transcription factor, AP2α/Tfap2α [39], activating enhancer binding protein 2 alpha factor, Jun/c-Jun [40, 41], a component of the AP-1 early response transcriptional complex, Etv5/Erm [42], an ets-domain transcription factor, Pax3 [43, 44], a paired homeodomain protein, and Nfatc4 [45], nuclear factor of activated T cells.

Through these studies, we identified distinct expression profiles for NCCs, NCC precursors, SCPs, iSCs, pro-myelinating SCs and mature myelinating/non-myelinating SCs, and demonstrated an underlying heterogeneity of the SC pool. Our findings also demonstrated that the de-differentiated SC is a unique SC subtype, distinct from any one developmental stage in the SC lineage. Given that this ‘repair’ SC subtype is the driving force behind efficient regeneration in uncompromised nerve injuries, methods to recapitulate this phenotype could be further investigated as a therapeutic avenue to treat chronic nerve injury and demyelinating disease.

## Materials and Methods

### Animals

CD1, C57/BL6, and Sox2eGFP mice [46] mice were purchased from Charles River Laboratories (Senneville, QC) and Jackson Laboratory (ME, United States) and maintained in a 12 hr light cycle. Embryos were staged using the morning of the vaginal plug as embryonic day (E) 0.5. Pregnant females were housed individually after mating, and euthanized using cervical dislocation for embryo collection. Adult mice used for peripheral nerve harvesting were group housed before injury, and then singly housed with enrichment post-injury. These animals were euthanized using an overdose (0.1mL) of Sodium Pentobarbital (54.7mg/mL, Ceva Sante Animale). Animal procedures were approved by the University of Calgary Animal Care Committee in compliance with the Guidelines of the Canadian Council of Animal Care.

### Embryo processing

Whole embryos were collected for stages E9.0 and E10.5, while only bodies were collected for stages E12.5, E14.5 and E18.5. For post-natal studies, sciatic nerves were harvested from the limbs of P7/P65 pups. The embryos and nerves were fixed in 4% paraformaldehyde (PFA)/1X diethyl-pyrocarbonate (DEPC) treated phosphate-buffered saline (PBS) for ~ 4–20 hours at 4°C. The embryos and nerves were rinsed in DEPC-PBS and transferred to 20% sucrose/1X DEPC-PBS, and were kept overnight at 4°C. The embryos and nerves were then embedded using O.C.T™ (Tissue-Tek®, Sakura Finetek U.S.A. Inc., Torrance, CA) and stored at -80°C. For the injury study, five days after injury (P65) mice were sacrificed by overdose of sodium pentrobarbital (i.p.; CEVA, Sante Animale). Nerves were removed and fixed in 4% PFA for two hours and subsequentally placed in 30% sucrose overnight. The next day, nerves were embedded in O.C.T.™ compound, frozen on dry ice and stored at -80°C before cutting cryosections on a Leica cryostat (Richmond Hill, ON).

### Surgery

For crush injury, P60 mice were anesthetized using isofluorane (5% induction and 2% maintenance) and then given a preoperative subcutaneous injection of 0.1 mL (0.03 mg/mL) buprenorphine. Hindlimbs were shaved and then cleaned twice with 70% EtOH followed by 10% providine iodine. On only the right hindlimb, the sciatic nerve was crushed at mid-thigh using #10 forcep for one minute. Muscle and skin were sutured back together (7–0 Prolene and 7–0 Silk, black braided; Ethicon Inc.) and buprenorphine was administered once a day for 4 days following surgery.

### Immunohistochemistry

Transverse 10 μm cryosections of the embryonic trunk and longitudinal 13 μm nerve sections were collected on SuperFrost™ Plus slides (Thermo Scientific). Tissue sections were washed and permeabilized in 1x PBS/0.1% TritonX (PBT) followed by blocking with 10% normal horse serum/PBT (blocking solution) for ~1 hour at room temperature. Prior to blocking, antigen epitope retrieval was carried out by heating sections in sodium citrate buffer for 20 minutes in a microwave. Sections were then allowed to cool down, washed with PBT and then blocked as above. Primary antibodies were diluted in blocking solution and were added to the sections and kept overnight at 4°C. Sections were washed with PBT and secondary antibody diluted 1/500 in PBT was applied for 1hour at room temperature. Sections were then washed and counterstained with 4′,6-diamidino-2-phenylindole (DAPI; Santa Cruz Biotechnology) diluted in PBT (1/5000) or Hoechst 33258 (Sigma-Aldrich #14530; 1:500) at room temperature. Sections were washed in PBS and mounted with coverslips using AquaPolymount (Polysciences). Primary antibodies included: rabbit anti-AP2α (Abcam ab52222; 1:200), rabbit anti-Egr1 (Aviva Systems Biology ARP32241_P050; 1:200), rabbit anti-Egr2 (Bioss Antibodies bs-8368R; 1:200, Santa Cruz Biotechnology sc-20690; 1:200), mouse anti-Egr2 (Abcam ab168771; 1:50), rabbit anti-Etv5 (Abcam ab102010; 1:300), rabbit anti-Jun (Abcam ab31419; 1:300), rabbit anti-Ki67 (Vector Laboratories #VP-K451), rabbit anti-Nfatc4 (Abcam ab3447; 1:200), mouse anti-NeuN (Millipore MAB377; 1:200), goat anti-Oct6 C-20 (Santa Cruz Biotechnology sc-11661; 1:50), mouse anti-Pax3 (Developmental Studies Hybridoma Bank; 1:5), rabbit anti-Sox2 (Cell Signaling #3728; 1:200), rabbit anti-Sox9 (Millipore AB5535; 1:500), goat anti-Sox10 (Santa Cruz Biotechnology sc-17343; 1:400), rabbit anti-Sox10 (Millipore AB5727; 1:200), and rabbit anti-Yy1 (Abcam ab12132; 1:200). For Pax3, Sox9, and Oct6, serial immunostaining was conducted. Secondary antibodies included: Alexa 568 donkey anti-rabbit, Alexa 488 donkey anti-goat, Alexa 555 donkey anti-mouse, Alexa 647 donkey anti-goat, Alexa 488 donkey anti-rabbit, Alexa 555 donkey anti-rabbit, Alexa 488 doney anti-mouse (Invitrogen), and FluoroMyelin (LifeTechnologies) and were diluted in PBT at 1 in 500.

### RNA *in situ* hybridization

RNA *in situ* hybridization was performed as previously described [47].

### Microscopy and image processing

For the embryonic and P7 sections, images were captured with a QImaging RETIGA 2000R or QImaging RETIGA EX digital camera and a Leica DMRXA2 optical microscope using OpenLab5 software (Improvision; Waltham MA). P65 nerve sections (injured and un-injured) were imaged using an inverted epifluorescent microscope (40x objective with oil, z-stack, Axio Observer Research Microscope; Zeiss Observer.Z1). Negative controls were included to distinguish non-specific secondary antibody binding. The captured images were processed using Adobe Photoshop software. Quantification in the embryonic studies was restricted to the Sox10^+^ NCCs at E9.0, and Sox10^+^ cells populating the dorsal and ventral root and DRG, and the exiting spinal nerve at the remaining stages. Quantification in the injury studies was conducted using a minimum of three images taken distal to the crush site from three different tissue sections for each animal (n = 3 mice per group). Double and single-positive cells (protein of interest co-localizing with Sox10^+^ SCs and Sox10^+^ SCs) were manually counted using Adobe Photoshop software. An unpaired student’s t-test was performed using Prism software (GraphPad) to determine whether there was a difference in protein expression of intact or actuely injured adult nerves (significance p<0.05).

## Results

**Fig 1:**
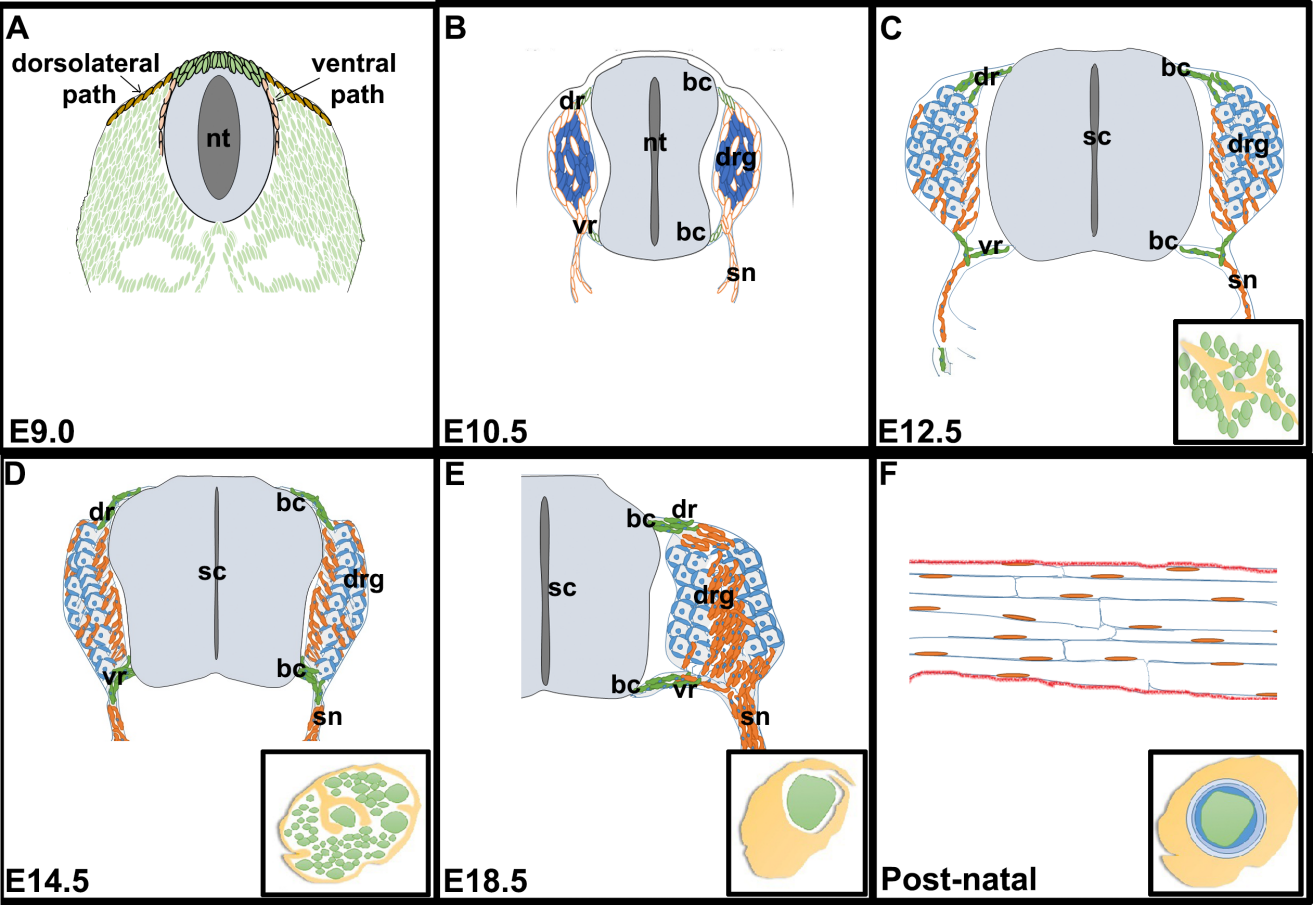
Schematic representation of the different phases of Schwann cell development. Schematic images of transverse sections through the trunk of E9.0 (A), E10.5 (B), E12.5 (C), E14.5 (D) and E18.5 (E) embryos, and a longitudinal section through the postnatal sciatic nerve (F). Insets in C-F show transverse sections through the sciatic nerve. bc, boundary cap; dr, dorsal root; drg, dorsal root ganglia; nt, neural tube; sn, spinal nerve; vr, ventral root.

To perform a temporal assessment of the developmental expression profiles of transcription factors implicated in mouse SC lineage progression, we analysed five embryonic stages of development (i.e., E9.0, E10.5, E12.5, E14.5, and E18.5) as well as the postnatal nerve (P7 and P65). These stages are associated with distinct phases of SC development: (i) delamination of trunk NCCs from the dorsal neural tube (E9.0); (ii) migration of trunk NCCs along the ventral path to populate the developing DRG and peripheral nerves (E10.5); (iii) association of SCPs with developing axons (E12.5); (iv) maturation of SCPs into iSCs (E14.5); (v) differentiation of a subset of iSCs into pro-myelinating SCs (E18.5) and (vi) terminal differentiation of iSCs into myelinating and non-myelinating SCs (P7/P65) [48] (Fig 1A–1F).

### Expression of Schwann cell lineage markers in neural crest cells

**Fig 2:**
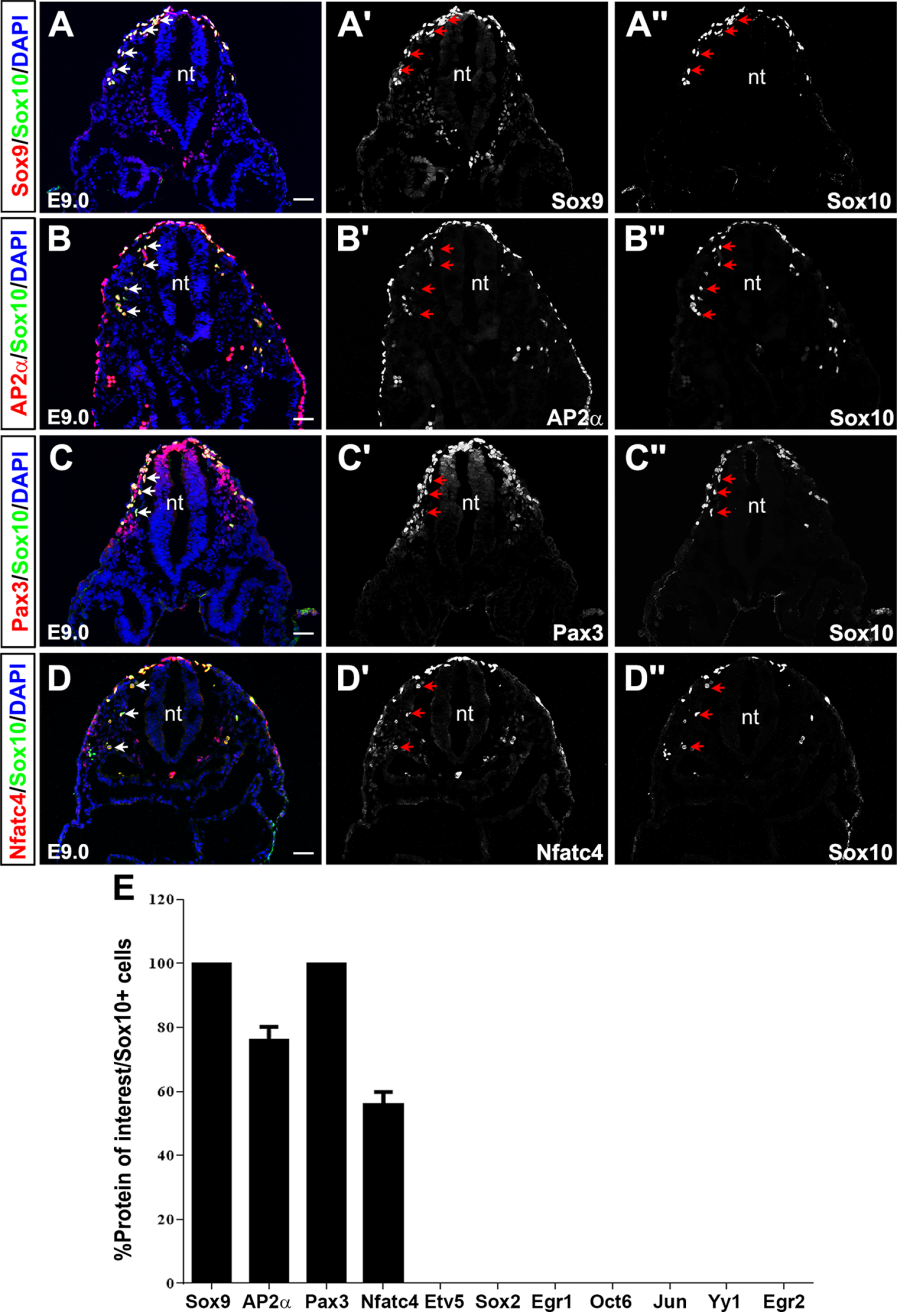
Expression of SC lineage markers in E9.0 NCCs. (A-E,A'-D',A''-D'') Co-expression of Sox10 with Sox9 (A-A''), AP2α (B-B''), Pax3 (C-C''), Nfatc4 (D-D'') in E9.0 NCCs. A-D are merged images of Sox10 (green) and protein of interest (red). Blue is DAPI counterstain. A'-D' shows expression profiles of the protein of interest, while A''-D'' show Sox10 expression. Arrows indicate co-expression of protein of interest with Sox10 in NCCs taking the ventral migratory pathway. Quantification of percent Sox10^+^ co-expression with each of the proteins of interest (E). Error bars = S.E.M. nt, neural tube. Scale bars, 40μm.

We first assessed the expression of SC markers in delaminating trunk NCCs at E9.0 (Fig 1A). To reliably label NCCs, and at later stages, to mark peripheral cells fated for the glial lineage, we used Sox10 as a co-label in all marker studies. Sox10 is continually expressed in NCCs, SCs and satellite glia throughout development [26, 28], and is required for the differentiation of all peripheral glia [28, 49, 50]. At E9.0, Sox10 was expressed in trunk NCCs delaminating from the neural tube, including those following both dorsolateral and ventral migratory routes (Fig 2A–2D and 2A''–2D''). In the E9.0 ventral migratory pathway, Sox10 was strongly co-expressed with Sox9 (100±0% Sox9^+^Sox10^+^/Sox10^+^ cells; Fig 2A–2A'' and 2E), AP2α (76.1±4.1% AP2α^+^Sox10^+^/Sox10^+^ cells; Fig 2B–2B'' and 2E), Pax3 (100±0% Pax3^+^Sox10^+^/Sox10^+^ cells; Fig 2C–2C' and 2E'), and Nfatc4 (56.0±3.8% Nfatc4^+^Sox10^+^/Sox10^+^ cells; Fig 2D–2D'' and 2E). These observations were consistent with previous reports documenting the activation of a Nfat transcriptional reporter [45] and the expression of Sox9 [26], AP2α [39], and Pax3 [51] in migrating NCCs. Of these markers, Sox9 induces a NCC phenotype [25], and its expression biases migrating NCCs towards glial and melanocyte lineage selection [26], whereas essential roles have only been documented at later developmental stages for the remaining transcription factors in the SC lineage: AP2α maintains a SCP fate, impeding the transition to an iSC [39], Pax3 regulates SC proliferation [52] and Nfatc4 acts synergistically with Sox10 to initiate Egr2 expression in SCs [45].

At E9.0, Etv5 was also expressed, but only in a very small number of Sox10^+^ NCCs (S1A–S1C Fig), which may be why previous reports have suggested that Etv5 is not expressed in the E9.0 neural crest [42]. Jun expression was also detected in a subset of NCCs, but instead of labeling cells in the ventral migratory pathway, it was primarily expressed in NCCs following a dorsolateral migratory route, which are pre-destined to a melanocyte fate (S1D–S1F Fig). In contrast, we did not detect the expression of Oct6 (S1G–S1I Fig), Sox2 (S1J–S1L Fig), Yy1 (S1M–S1O Fig), Egr1 (S1P–S1R Fig) or Egr2 (S1S–S1U Fig) in E9.0 trunk NCCs.

Thus, Sox9, AP2α, Pax3, and Nfatc4 are widely co-expressed with Sox10 in E9.0 NCCs following a ventral migratory route, albeit not in all SCs for AP2α and Nfatc4, whereas Etv5 is only expressed in a small subset of NCCs, and Jun instead marks dorsolaterally migrating NCCs.

### Expression of Schwann cell lineage markers in migratory NCC precursors

By E10.5, NCCs have coalesced to form the DRG, which at this stage, are comprised of sensory neurons and migratory NCC precursors that are destined to become SCPs and satellite glial cells (Fig 1B). Emanating from the DRG are the dorsal and ventral roots, which coalesce to form the mixed sensory/motor spinal nerve. NCCs also give rise to a subset of multipotent cells called boundary cap cells that are located at the dorsal root entry zone and motor exit points. SCs populating the dorsal and ventral roots find their origin in these boundary cap cells, as do a few satellite glial cells [15].

**Fig 3:**
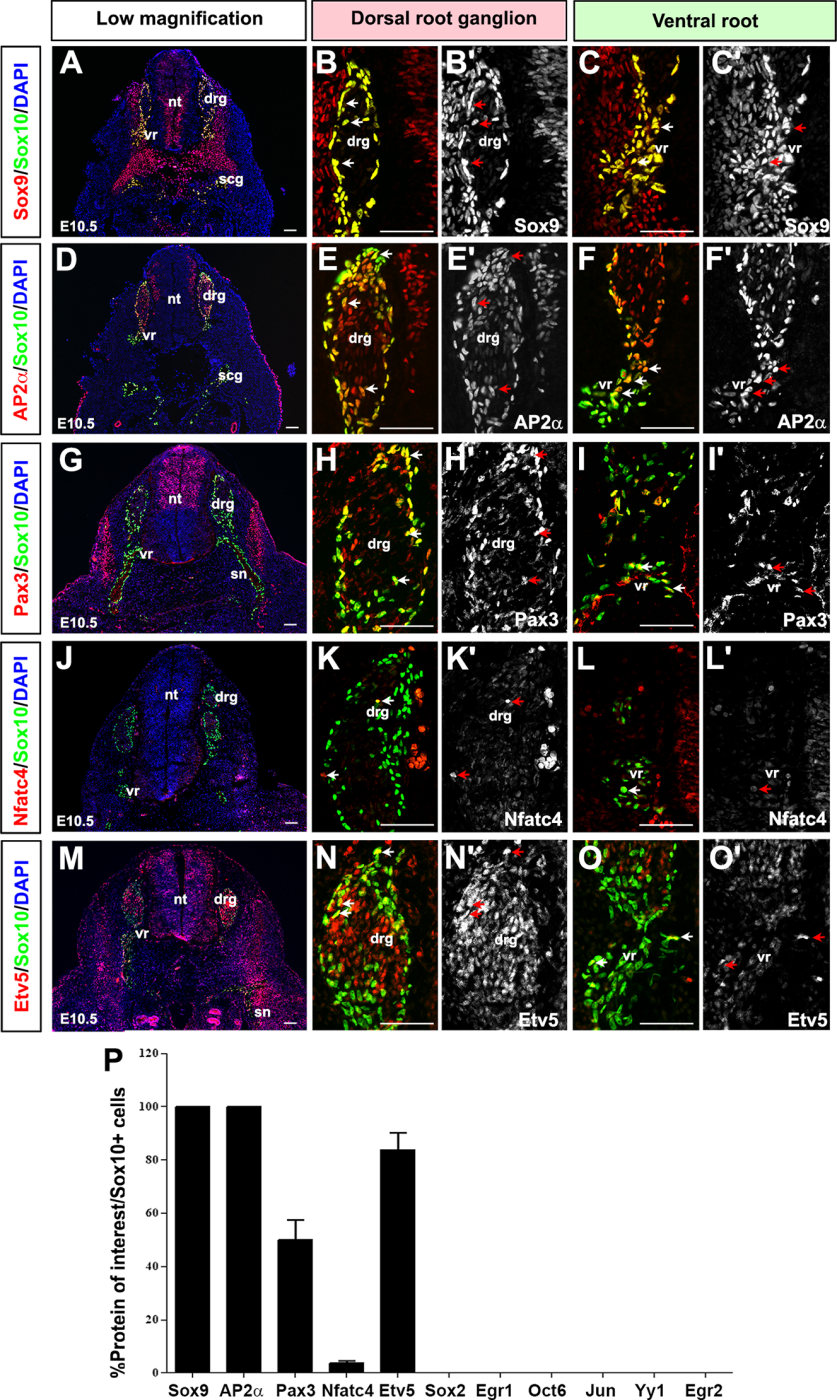
Expression of SC lineage markers in E10.5 NCC precursors. (A-P) Co-expression of Sox10 with Sox9 (A-C'), AP2α (D-F'), Pax3 (G-I'), Nfatc4 (J-L'), and Etv5 (M-O') in transverse sections through the E10.5 trunk. Merged images of the protein of interest (red) with Sox10 (green) (A,D,G,J,M). Blue is DAPI counterstain. High magnification images of the DRG, showing merged images of the protein of interest (red) and Sox10 (green) (B,E,H,K,N), and single protein of interest images in white (B',E',H',K',N'). High magnification images of the ventral roots, showing merged images of the protein of interest (red) and Sox10 (green) (C,F,I,L,O), and single protein of interest images in white (C',F',I',L',O'). Arrows mark co-expression of proteins of interest with Sox10 in NCC precursors. Quantification of percentage of Sox10^+^ cells that co-express each of the proteins of interest (P). Error bars = S.E.M. drg, dorsal root ganglion; nt, neural tube; scg, sympathetic chain ganglion; sn, spinal nerve; vr, ventral root. Scale bars, 60μm.

At E10.5, Sox10 was expressed in boundary cap cells in the dorsal and ventral roots of the DRG, in presumptive SCPs, in satellite glia in the periphery of the DRG, and in migratory NCCs in the spinal nerve (Fig 3A–3C). Sox10 expression was for the most part excluded from the central DRG, where NeuN^+^ neuronal cells are located (S2D and S2E Fig). Similar to their co-expression profiles in E9.0 NCCs, Sox10 was largely co-expressed with Sox9 (100±0% Sox9^+^Sox10^+^/Sox10^+^ cells; Fig 3A–3C' and 3P) and AP2α (100±0% AP2α^+^Sox10^+^/Sox10^+^ cells; Fig 3D–3F' and 3P). Importantly, not all Sox9^+^ cells were Sox10^+^, as Sox9 was expressed in a larger subset of non-glial cells (as seen in other stages as well), confirming that the antibodies are not recognizing epitopes shared between Sox family members. We further confirmed the specificity of the antibodies by demonstrating that Sox9 and Sox10 have distinct staining patterns in the CNS (S3A–S3F Fig). Pax3 also continued to be co-expressed with Sox10, however at reduced levels (50.1±7.4% Pax3^+^Sox10^+^/Sox10^+^ cells; Fig 3G–3I' and 3P), and a much smaller number of Sox10^+^ NCCs coalescing in the E10.5 DRG and in the ventral root expressed Nfatc4 (3.7±0.8% Nfatc4^+^Sox10^+^/Sox10^+^ cells; Fig 3J–3L' and 3P). In addition, Etv5 expression was initiated at E10.5 in Sox10^+^ NCC precursors in the DRG (83.9±6.3% Etv5^+^Sox10^+^/Sox10^+^ cells; Fig 3M–3O' and 3P), consistent with previous reports [42, 53].

*In vitro* studies suggested that a block of *Etv5* function in NCCs affects neuronal and not glial fate specification [54]. Consistent with these findings, Etv5 (S2J–S2L Fig) as well as AP2α (S2G–S2I Fig) were also expressed in the neuronal-rich central part of the DRG, where they were co-labeled with NeuN, a pan-neuronal marker. In contrast, Sox9 (Fig 3A–3C) was exclusively co-expressed with Sox10 in the DRG periphery, where presumptive peripheral glia are located. Sox9 was also expressed with Sox10 in the ventral root, developing spinal nerve, and in mesenchymal tissue between the DRG and somites (Fig 3A–3C). In contrast, Oct6 (S4A–S4E Fig), Jun (S4F–S4J Fig), Sox2 (S4K–S4O Fig), Yy1 (S4P–S4T Fig), Egr1 (S4U–S4Y Fig) and Egr2 (S4Z–S4DD Fig) expression was not detectable in the developing PNS at this stage, including in Sox9^+^ and Sox10^+^ glial cells.

In summary, at E10.5, Sox9, AP2α, Pax3, Nfatc4 and Etv5 are co-expressed with Sox10 in presumptive NCC-derived glial cells in the dorsal and ventral roots, DRG and developing spinal nerve, and a subset of these cells have progressed to a SCP fate based on the co-expression of glial markers (data not shown).

### Expression of Schwann cell lineage markers in Schwann cell precursors

By E12.5, the vast majority of NCC precursors destined for a glial lineage have differentiated into either SCPs or satellite glia. Morphologically, SCPs are distinguished from migrating NCCs as they associate directly with growing axon bundles, but they lack the basal lamina secreted by iSCs (Fig 1C). SCPs are located proximal to the growing nerve tip, and they participate in compacting the nerves while also guiding axons to their targets [10]. Satellite glia can be partially distinguished from SCPs based on their location; satellite glia are in the DRG but are excluded from the nerves, whereas SCPs are found in both locations. However, because of the lack of specific markers, satellite glial cells are not easily distinguished from SCPs within the DRG, although they do have a more flattened nuclear morphology [55, 56]. For simplicity, we use the SCP nomenclature for precursor cells for both satellite glia and SCs.

**Fig 4:**
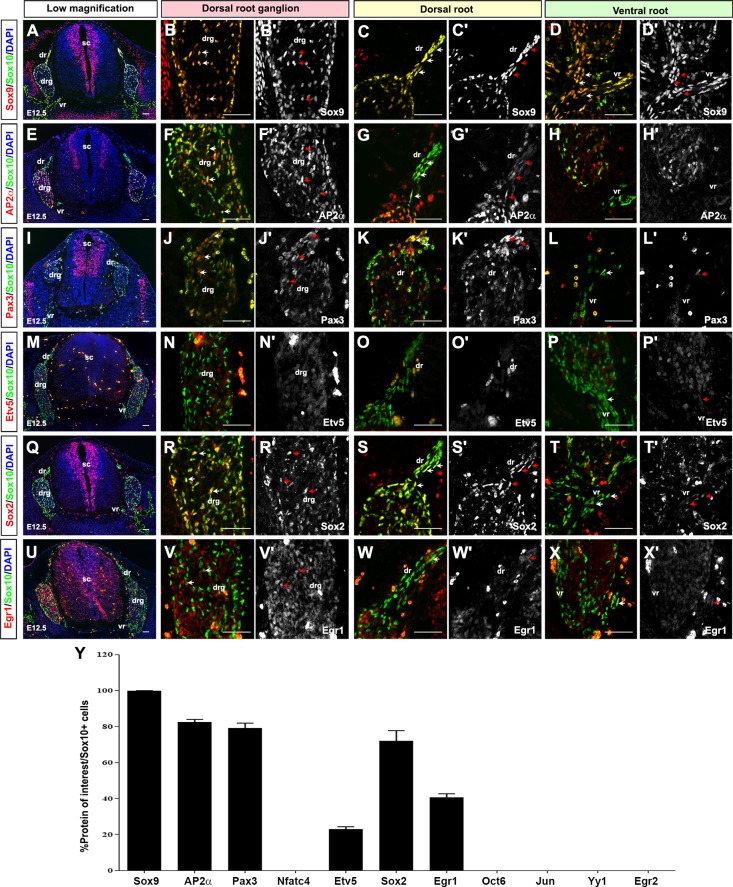
Expression of SC lineage markers in E12.5 SCPs. (A-Y) Co-expression of Sox10 with Sox9 (A-D'), AP2α (E-H'), Pax3 (I-L'), Etv5 (M-P'), Sox2 (Q-T'), and Egr1 (U-X') in transverse sections through the E12.5 trunk. Low magnification merged images of protein of interest (red) and Sox10 (green) (A,E,I,M,Q,U). Blue is DAPI counterstain. High magnification images of the DRG, showing merged images of the protein of interest (red) and Sox10 (green) (B,F,J,N,R,V), and single protein of interest images in white (B',F',J',N',R',V'). High magnification images of the dorsal roots, showing merged images of the protein of interest (red) and Sox10 (green) (C,G,K,O,S,W), and single protein of interest images in white (C',G',K',O',S',W'). High magnification images of the ventral roots, showing merged images of the protein of interest (red) and Sox10 (green) (D,H,L,P,T,X), and single protein of interest images in white (D',H',L',P',T',X'). Arrows indicate co-expression of proteins of interest with Sox10 in SCPs. Quantification of percentage of Sox10^+^ cells that co-express each of the proteins of interest (Y). Error bars = S.E.M. dr, dorsal root; drg, dorsal root ganglion; sc, spinal cord; scg, sympathetic chain ganglion; sn, spinal nerve; vr, ventral root. Scale bars, 60μm.

At E12.5, Sox10 expression was slightly more widespread in the DRG compared to E10.5, marking both peripheral and central DRG cells (Fig 4A and 4B). The extension of Sox10 expression into the central DRG did not include sensory neurons, as Sox10 was not co-expressed with NeuN at this stage (S5D–S5F Fig). Instead, Sox10 was expressed exclusively in presumptive peripheral glia, as previously suggested [26, 28]. Sox10 was also expressed in the dorsal (Fig 4A and 4C) and ventral (Fig 4A and 4D) roots, where migrating SCPs are located. In co-expression studies, Sox10 continued to be highly co-expressed with Sox9 (99.8±0.2% Sox9^+^Sox10^+^/Sox10^+^ cells; Fig 4A–4D'' and 4Y) and AP2α (82.3±1.6% AP2α^+^Sox10^+^/Sox10^+^ cells; Fig 4E–4H' and 4Y), whereas Pax3 co-expression started to decline slightly (79.1±2.8% Pax3^+^Sox10^+^/Sox10^+^ cells; Fig 4I–4L' and 4Y). A larger drop was seen in the number of Sox10^+^ precursor cells that co-expressed Etv5 (22.9±1.5% Etv5^+^Sox10^+^/Sox10^+^ cells; Fig 4M–4P' and 4Y). Most Sox10^+^Etv5^+^ cells lined the periphery of the DRG and were likely satellite glial cells based on their flattened nuclei. Etv5 expression was also detected in a few SCPs in the dorsal and ventral root. In addition, Etv5 was co-expressed with NeuN^+^ in DRG sensory neurons (S5M–S5O Fig). This data is consistent with previous reports indicating that *Etv5* transcripts are detected in satellite glial cells and DRG sensory neurons [42, 53], although this previous study did not detect *Etv5* transcripts in the sciatic nerve [54]. In contrast to Etv5, Sox9 (S5A–S5C Fig) and AP2α (S5G–S5I Fig) were not co-expressed with NeuN in DRG sensory neurons and were thus glial-specific.

At E12.5, Sox2 and Egr1 expression was also initiated in a subset of Sox10^+^ SCPs (71.9±5.8% Sox2^+^Sox10^+^/Sox10^+^ cells; Fig 4Q–4T' and 4Y, 40.7±0.8% Egr1^+^Sox10^+^/Sox10^+^ cells; Fig 4U–4X' and 4Y) in both the DRG and roots. In contrast, Nfatc4 (S6A–S6G Fig) was no longer co-expressed with Sox10 at E12.5. Additional transcription factors that were not co-expressed with Sox10 in E12.5 SCPs included Oct6 (S6H–S6N Fig), Jun (S6O–S6U Fig), Yy1 (S6V–S6BB Fig) and Egr2 (S6CC–S6II Fig).

In summary, E12.5 SCPs undergo a temporal shift in their expression profile, retaining the expression of Sox10, Sox9, AP2α and Pax3, as observed in E10.5 NCCs, while losing the expression of Nfatc4 and Etv5, and gaining the expression of Sox2 and Egr1. The initiation of Sox2 (Fig 4Q–4T') and Egr1 (Fig 4U–4X') expression in E12.5 peripheral glia is consistent with previous reports documenting the expression of Sox2 in SCPs and iSCs [23] and Egr1 in SCPs [35]. However, Egr1 is also co-expressed with NeuN in the DRG (S5S–S5U Fig), indicating that it also labels sensory neurons. Of the genes that are newly expressed at this stage, Sox2 expression is upregulated in a subset of cells that develop into the PNS [23], and within the SC lineage, Sox2 regulates the differentiation of SCPs into myelinating SCs versus melanocytes [24], while Egr1 is considered a non-myelinating SC marker [35].

### Expression of Schwann cell lineage markers in immature Schwann cells

As development proceeds, SCPs can either give rise to iSCs, or alternatively, endoneurial fibroblasts and melanocytes [18, 19]. iSCs appear from E14.5 and persist until just before birth [10] (Fig 1D). iSCs cluster around several axons and deposit a basal lamina that surrounds both the iSCs and the axonal bundle [20]. iSCs then penetrate axonal bundles, positioning larger diameter axons in the periphery for radial sorting. A characteristic feature of iSCs is that they secrete autocrine survival factors so that they are no longer entirely dependent on axon-derived Neuregulin1, present on the surface of axons [10, 57].

**Fig 5:**
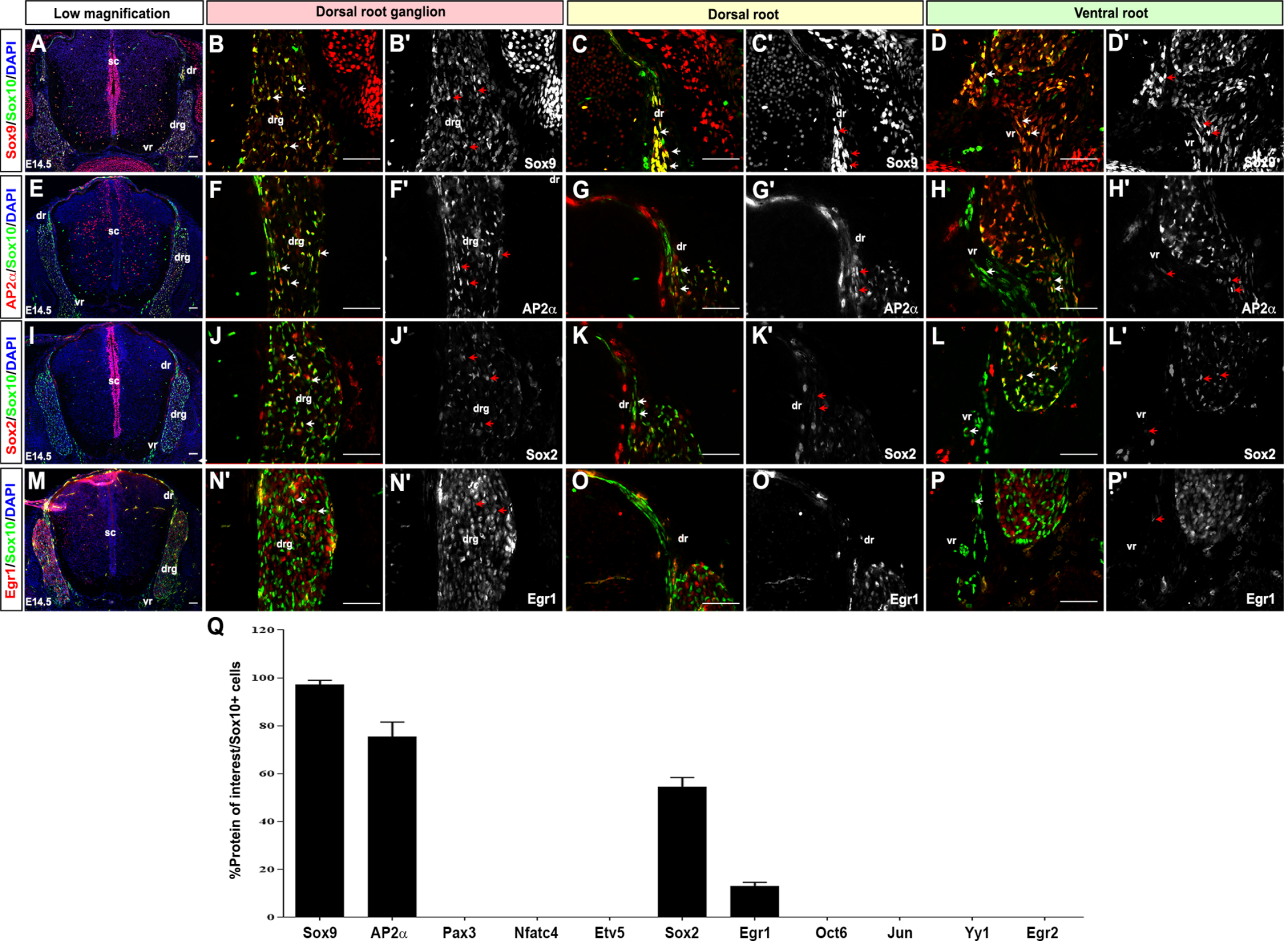
Expression of SC lineage markers in E14.5 iSCs. (A-Q) Co-expression of Sox10 with Sox9 (A-D'), AP2α (E-H'), Sox2 (I-L') and Egr1 (M-P') in transverse sections through the E14.5 trunk. Low magnification merged images of protein of interest (red) and Sox10 (green) (A,E,I,M). Blue is DAPI counterstain. High magnification images of the DRG, showing merged images of the protein of interest (red) and Sox10 (green) (B,F,J,N), and single protein of interest images in white (B',F',J',N'). High magnification images of the dorsal roots, showing merged images of the protein of interest (red) and Sox10 (green) (C,G,K,O), and single protein of interest images in white (C',G',K',O'). High magnification images of the ventral roots, showing merged images of the protein of interest (red) and Sox10 (green) (D,H,L,P), and single protein of interest images in white (D',H',L',P'). Arrows indicate co-expression of proteins of interest with Sox10 in iSCs. Quantification of percentage of Sox10^+^ cells that co-express each of the proteins of interest (Q). Error bars = S.E.M. dr, dorsal root; drg, dorsal root ganglion; sc, spinal cord; vr, ventral root. Scale bars, 60μm.

By E14.5, Sox10 was expressed in scattered cells throughout the DRG, including in the center and periphery, where iSCs and satellite glia are located (Fig 5A and 5B). Sox10 was not co-expressed with NeuN, confirming that it is exclusively labeling glial precursors at E14.5 (data not shown). In addition, Sox10 was expressed in the dorsal (Fig 5A and 5C) and ventral roots (Fig 5A and 5D) and in the exiting spinal nerve (data not shown). In co-expression studies at E14.5, Sox10 was still highly co-expressed with Sox9 (97.1±1.8% Sox9^+^Sox10^+^/Sox10^+^ cells; Fig 5A–5D' and 5Q) and AP2α (75.4±6.1% AP2α^+^Sox10^+^/Sox10^+^ cells; Fig 5E–5H and 5Q'). In contrast, a decline in Sox2 (54.4±1.6% Sox2^+^Sox10^+^/Sox10^+^ cells; Fig 5I–5L' and 5Q) and Egr1 co-expression with Sox10 was seen (12.8±0.7% Egr1^+^Sox10^+^/Sox10^+^ cells; Fig 5M–5P' and 5Q).

Compared to E12.5 SCPs, E14.5 iSCs also lost the expression of Pax3 (S7A–S7G Fig) and failed to express Nfatc4 (S7H–S7N Fig), Etv5 (S7O–S7U Fig), Jun (S7V–S7BB Fig), Oct6 (S7CC–S7II Fig), Yy1 (S7JJ–S7PP Fig) and Egr2 (S7QQ–S7WW Fig). Thus, the major difference between E12.5 SCPs and E14.5 iSCs is the loss of Pax3 expression. Previous studies had detected *Pax3* transcripts in SCPs as well as in iSCs, but indicated that *Pax3* transcript levels decline in late iSCs undergoing radial sorting, and protein levels were not assessed [43].

In summary, E14.5 iSCs are characterized by the expression of Sox10, Sox9, AP2α, Sox2 and Egr1, as well as glial lineage markers (data not shown), and they differ from E12.5 SCPs in that they no longer express Pax3.

### Expression of Schwann cell lineage markers in pro-myelinating Schwann cells

As iSCs develop, they extend cytoplasmic processes that penetrate axonal bundles, helping to distinguish large and small diameter axons. Larger axons are rearranged to the periphery of the bundle, with iSCs associating in a 1:1 proportional manner with these large diameter axons, resulting in radial sorting [20]. iSCs that associate with large diameter axons are a transient population termed pro-myelinating SCs; these are the SCs that will progress towards the myelinating stage (Fig 1E). In addition, a subset of late iSCs persists at E18.5 in association with multiple smaller axons, which are destined to become non-myelinating SCs. Thus, pro-myelinating SCs represent a transient phase in the SC lineage, first appearing just prior to birth, and expanding greatly on the first postnatal day [16].

**Fig 6:**
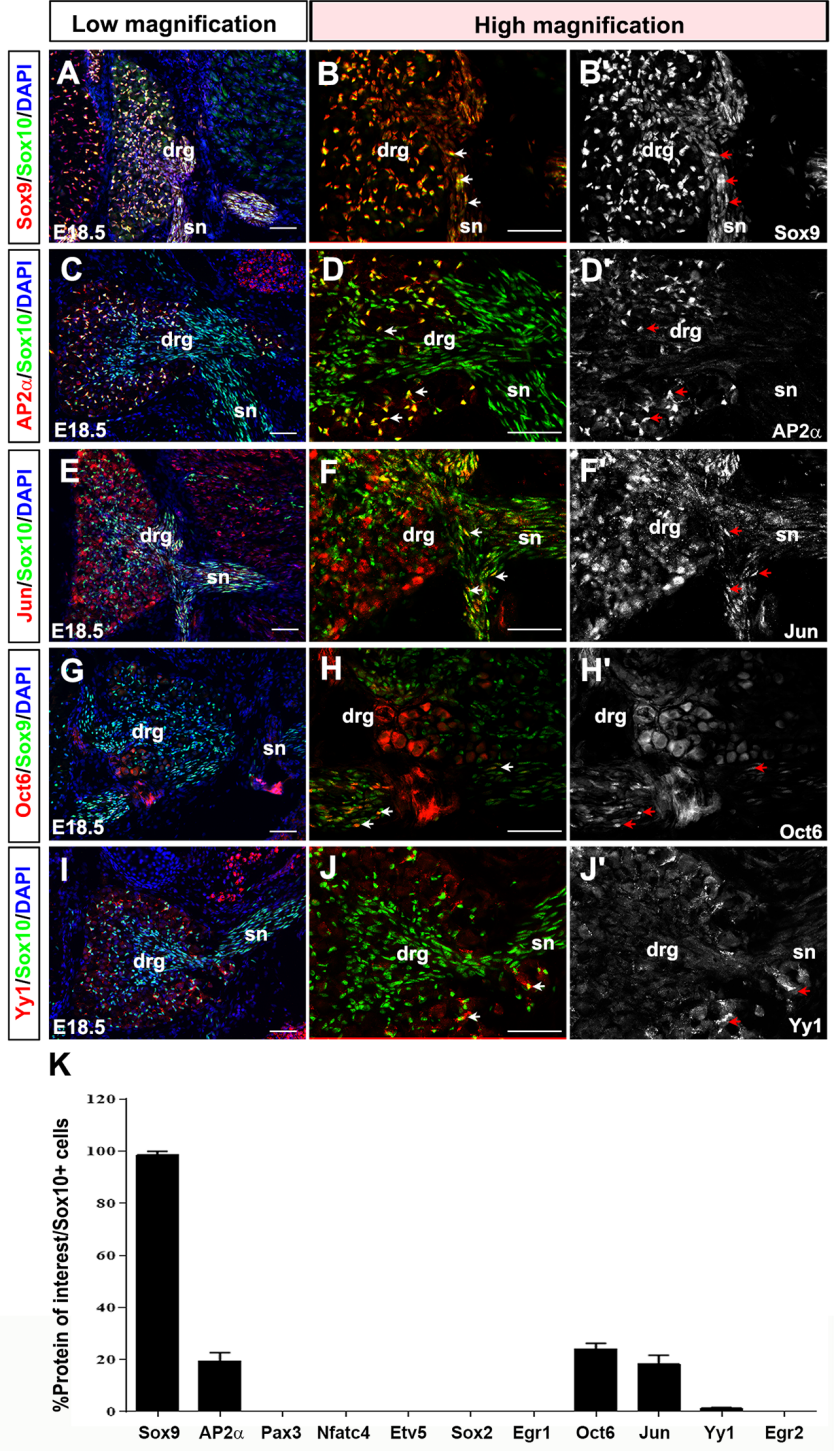
Expression of SC lineage markers in E18.5 late immature/pro-myelinating SCs. (A-K) Co-labeling of Sox10 with Sox9 (A-B'), AP2α (C-D'), Jun (E-F') and Yy1 (I-J'), and co-labeling of Sox9 and Oct6 (G-H') in transverse sections through the E18.5 trunk. Low magnification merged images of protein of interest (red) and Sox10 (green) (A,C,E,I) or Sox9 (G). High magnification images of the DRG, showing merged images of the protein of interest (red) and Sox10 (or Sox9) in green (B,D,F,H,J), and single protein of interest images in white (B',D',F',H',J'). Blue is DAPI counterstain in A-I. Arrows indicate co-expression of proteins of interest with Sox10 (or Sox9) in late immature and pro-myelinating SCs. Quantification of percentage of Sox10^+^ cells that co-express each of the proteins of interest (K). Error bars = S.E.M. drg, dorsal root ganglion; sc, spinal cord; sn, spinal nerve; vr, ventral root. Scale bars, 60μm.

At E18.5, Sox10 was expressed throughout the DRG, including in the center and periphery (Fig 6A and 6B). In the DRG center, Sox10^+^ late iSCs and pro-myelinating SCs amalgamated around the growing spinal nerve (Fig 6G and 6H). In dual labeling studies, the only transcription factors expressed at E14.5 that continued to be co-expressed with Sox10 at E18.5 were Sox9 (98.6±1.4% Sox9^+^Sox10^+^/Sox10^+^ cells; Fig 6A–6B' and 6K) and AP2α (19.4±3.2% AP2α^+^Sox10^+^/Sox10^+^ cells; Fig 6C–6D' and 6K). The number of AP2α^+^Sox10^+^ cells was greatly reduced and they were limited to the DRG and not detected in the nerves. Sox10^+^ late iSCs/pro-myelinating SCs also initiated the expression of Jun (18.3±3.3% Jun^+^Sox10^+^/Sox10^+^ cells; Fig 6E–6F' and 6K), Oct6 (24.1±2.1% Oct6^+^Sox9^+^/Sox9^+^ cells; Fig 6G–6H' and 6K), and Yy1 (1.3±0.3% Yy1^+^Sox10^+^/Sox10^+^ cells; Fig 6I–6J' and 6K) in a small number of cells.

Thus, three new transcription factors are expressed in E18.5 late iSCs and pro-myelinating SCs: Jun, Oct6 and Yy1. Jun has previously been shown to be expressed in late immature SCs and downregulated with the onset of myelination [41]. Oct6^+^ SCs were restricted for the most part to the boundary cap or the ventral root and exiting spinal nerve (Fig 6G–6H), most likely representing pro-myelinating SCs. Indeed, Oct6 is well studied for its role as a cell autonomous regulator of SC development [37] and a pro-myelinating SC marker [38], and is also essential for bringing about the pro-myelinating to myelinating SC transition [38]. Finally, Yy1 is important for attaining the myelination phenotype, such that conditional knockdown of Yy1 in SCs results in hypomyelinated nerves with poor expression of the myelin genes MPZ and Pmp22 [36].

At E18.5, Sox2 (S8G–S8I Fig) and Egr1 (S8M–S8O Fig) expression was lost, and Pax3 (S8A–S8C Fig), Etv5 (S8D–S8F Fig), Nfatc4 (S8J–S8L Fig), and Egr2 (S8P–S8R Fig) were also not expressed in E18.5 pro-myelinating SCs in either the DRG or nerve. The absence of Egr2 protein was surprising as *Egr2* transcripts have been detected in the embryonic SC lineage, most notably, in boundary cap cells, from early embryonic stages (S9Q–S9V Fig) [15]. One possibility is that *Egr2* is not translated. Indeed, all three of the Egr2 antibodies that we tested labeled SCs in adult nerves (S9F–S9H and S9N–S9P Fig); whereas Egr2 protein remained undetectable embryonically (S9A–S9E and S9I–S9M Fig).

In summary, E18.5 late iSCs and pro-myelinating SCs in the DRG and nerve roots are characterized by the expression of Sox10, Sox9, Jun, Oct6, Yy1 and AP2α (in the DRG only), and the loss of expression of Sox2 and Egr1.

### Expression of Schwann cell lineage markers in mature neonatal (P7) and adult (P65) myelinating and non-myelinating Schwann cells

**Fig 7:**
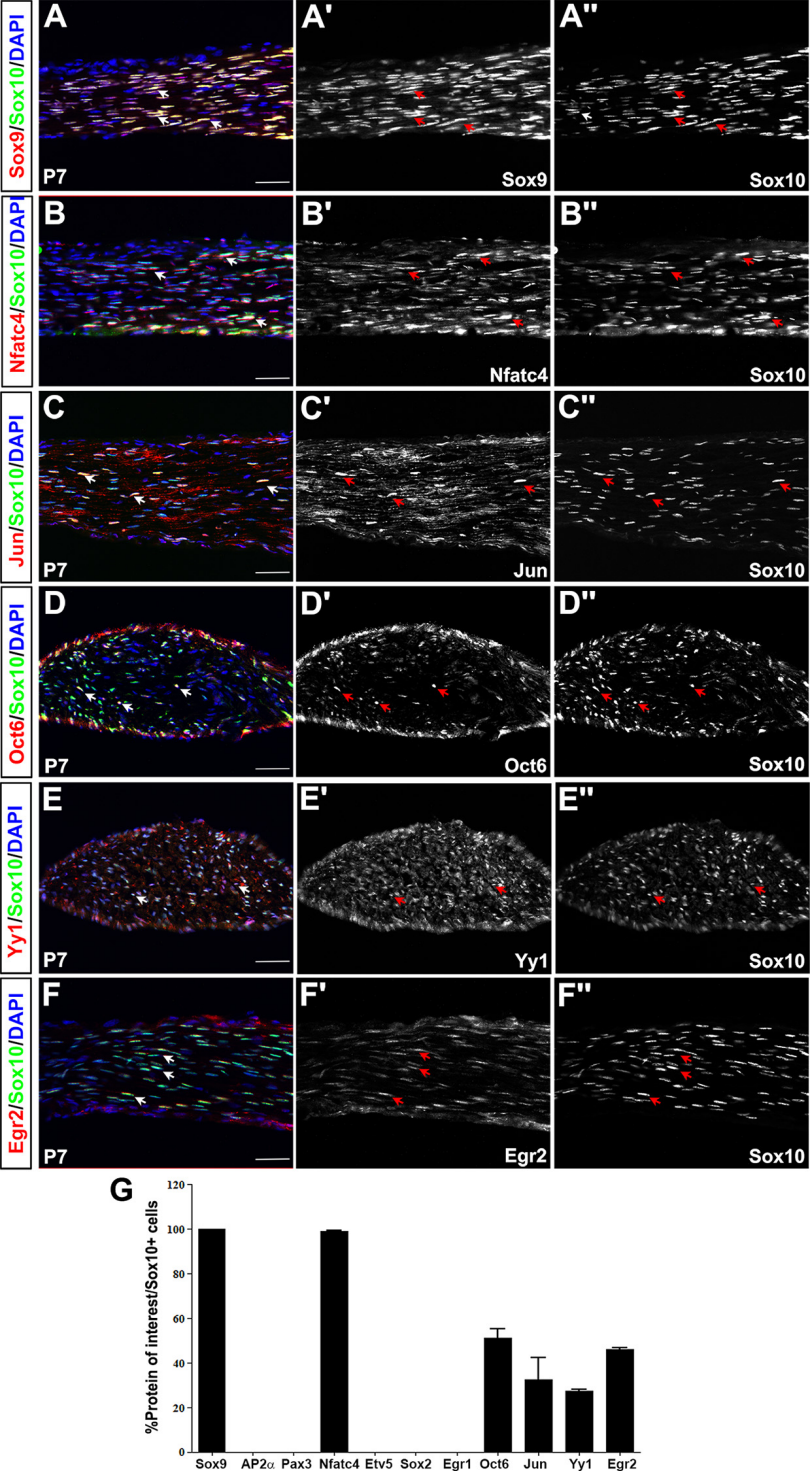
Expression of SC lineage markers in P7 mature SCs. (A-F'') Co-labeling of Sox10 with Sox9 (A-A''), Nfatc4 (B-B''), Jun (C-C''), Oct6 (D-D''), Yy1 (E-E''), and Egr2 (F-F'') in longitudinal sections of the P7 sciatic nerve. Merged images of the protein of interest in red and Sox10 in green (A-F). Blue is DAPI counterstain. Expression profiles of the protein of interest (A'-F') and Sox10 (A''-F''). Arrows indicate co-expression of proteins of interest with Sox10 in P7 mature SCs Quantification of percentage of Sox10^+^ cells that co-express each of the proteins of interest (G). Error bars = S.E.M. Scale bars, 40μm.

Pro-myelinating SCs that are in contact with larger diameter axons (with high levels of Neuregulin 1) progress to form myelinating SCs, whereas iSCs in association with smaller diameter axon bundles (releasing lower levels of Neuregulin1) post-radial sorting become non-myelinating SCs [21] (Fig 1F). At P7, Sox10^+^ myelinating and non-myelinating SCs surrounded the sciatic nerve (Fig 7A''–7F''). In co-labeling experiments in the P7 nerve, Sox10 was highly co-expressed with Sox9 (100±0% Sox9^+^Sox10^+^/Sox10^+^ cells; Fig 7A–7A'' and 7G) and Nfatc4 (99.1±0.5% Nfatc4^+^Sox10^+^/Sox10^+^ cells; Fig 7B–7B'' and 7G), Jun (32.6±9.6% Jun^+^Sox10^+^/Sox10^+^ cells; Fig 7C–7C'' and 7G), and to a lesser extent with Oct6 (51.3±3.9% Oct6^+^Sox10^+^/Sox10^+^ cells Fig 7D–7D'' and 7G), Yy1 (27.4±0.7% Yy1^+^Sox10^+^/Sox10^+^ cells; Fig 7E–7E'' and 7G) and Egr2 (46.1±0.7% Egr2^+^Sox10^+^/ Sox10^+^ cells; Fig 7F–7F'' and 7G). This expression profile was very similar to that observed in E18.5 pro-myelinating SCs, except that the expression of Nfatc4 was re-initiated and Egr2 protein was now detected at P7. In contrast, we did not detect the expression of AP2α (S10A–S10C Fig), Pax3 (S10D–S10F Fig), Etv5 (S10G–S10I Fig), Sox2 (S10J–S10L Fig), or Egr1 (S10M–S10O Fig) in the P7 nerve.

While Egr2 promotes the terminal differentiation of SCs to a myelinating phenotype, Egr1 and Pax3 are considered non-myelinating SC markers [35, 44]. Sox9 is also expressed later in neonatal myelinating and non-myelinating SCs [58], and can cooperatively bind the P0 promoter, a mature SC marker [30], suggesting that Sox9 may also function in postnatal SCs. Nfatc4 acts synergistically with Sox10 to activate the expression of Egr2 in the embryonic nerve [45, 59], which suggests a later role for Nfatc4 as Egr2 is required for the terminal differentiation of SCs to a myelinating phenotype [35]. Similarly, Oct6 [38] and Yy1 [36] are required for the myelination of peripheral nerves. In contrast, Jun expression is downregulated by Egr2 upon the onset of myelination, and is thus considered a marker of non-myelinating SCs [41].

Thus, the P7 nerve is primarily populated by Sox10^+^ myelinating SCs that co-express Sox9, Nfatc4, Jun, Oct6, Yy1 and Egr2, differing from E18.5 pro-myelinating SCs by the initiation of Nfatc4 and Egr2 protein expression. Non-myelinating SCs may also be present based on the expression of Jun, but they fail to express Egr1 (at least in the nucleus) and Pax3 at this stage.

**Fig 8:**
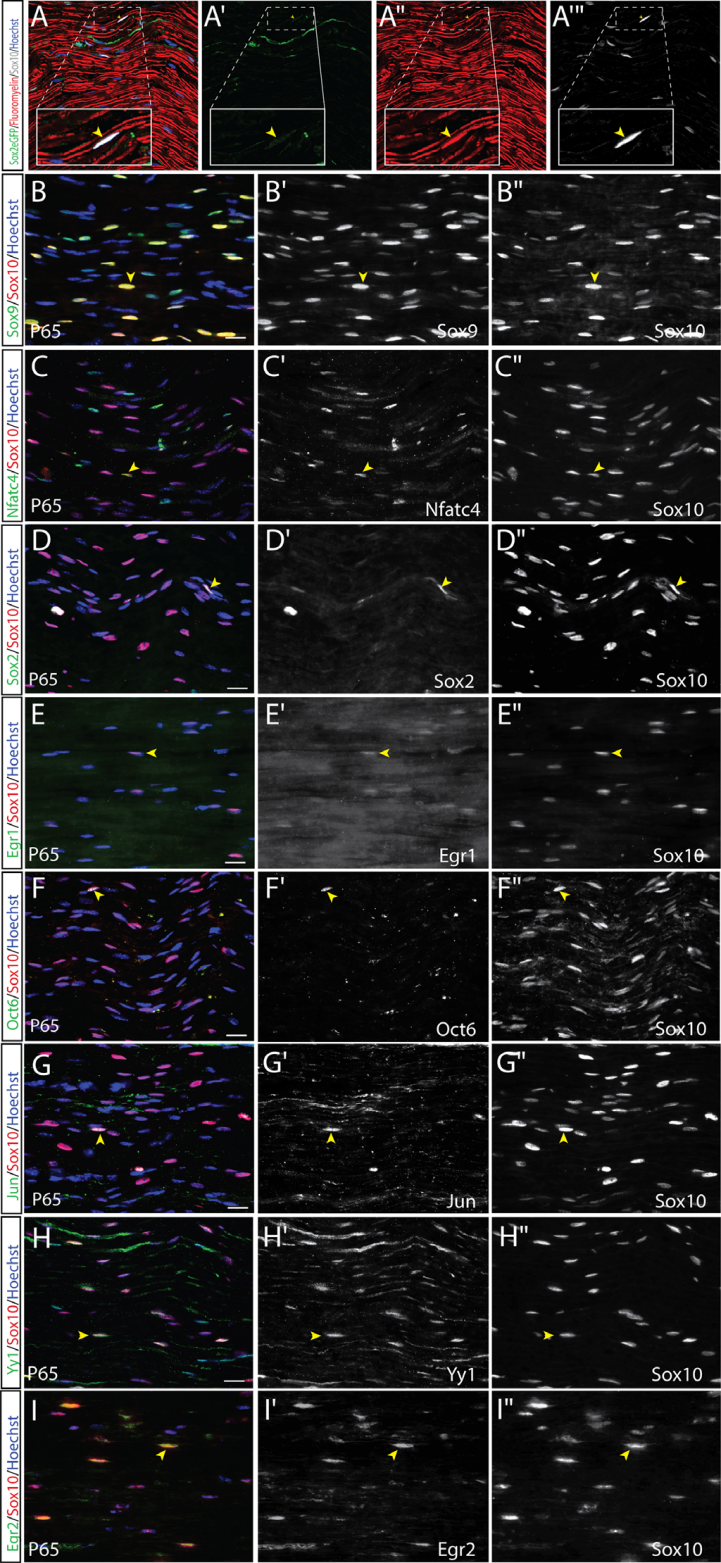
Expression of SC lineage markers in adult P65 uninjured sciatic nerve. (A-I'') Co-labeling of Sox10 with fluoromyelin (A-A‴), Sox9 (B-B''), Nfatc4 (C-C''), Sox2 (D-D''), Egr1 (E-E''), and Oct6 (F-F''), Jun (G-G''), Yy1 (H-H''), Egr2 (I-I'') in longitudinal sections of the uninjured P65 sciatic nerve. Merged images of protein of interest (green), Sox10 (red) and Hoechst (blue) (B-I). Yellow arrowheads indicate co-labelled cells. Scale bars, 20μm.

At P65, within the Sox10^+^ SC pool, 90.6±2.2% were associated with fluoromyelin^+^ myelin segments (Fig 8A–8A‴), indicative of a mature myelinating SC. A subset of Sox10^+^ cells were also observed in linear arrays independent of fluoromyelin^+^ myelin segments (not shown), and were presumed to be non-myelinating SCs associated with Remak bundles [48]. In addition, another subset of fluoromyelin-negative, Sox10^+^ SCs were associated with a GFP reporter driven by the Sox2 promoter (Fig 8A and 8A'), presumed to be rare iSCs [44] or non-myelinating SCs [60]. We restricted all marker co-localization analysis to Sox10^+^ cells that were independently localized (i.e., not associated with linear arrays) and were associated with myelin. The majority of Sox10^+^ SCs co-expressed Sox9 (88.4±0.3%; Fig 8B–8B''), Nfatc4 (53.3±6.8%; Fig 8C–8C''), Yy1 (50.0±0.6%; Fig 8H–8H''), and Egr2 (91.6±1.3%; Fig 8I–8I''). Egr1 expression was reinitiated in the Sox10^+^ SCs at P65 (27.1±13.6%; Fig 8E–8E''). The expression of both Egr1 and Egr2 in the P65 nerve suggests that both myelinating and non-myelinating SCs are present. Notably, Jun was only rarely expressed in Sox10^+^ SCs (0.6±0.5%; Fig 8G–8G''), as was Oct6 (4.3±1.8%; Fig 8F–8F''). Finally, consistent with a prior study [61], very few Sox2^+^Sox10^+^ SCs were observed within the adult nerve (4.3±2.9%; Fig 8D–8D''). Thus, the expression profile at P65 was very similar to that observed in P7 SCs, except for expression of Egr1 and appearance of rare Sox2^+^Sox10^+^ SCs. We did not detect the expression of AP2α (S11A–S11C Fig), Pax3 (S11G–S11I Fig), or Etv5 (S11M–S11O Fig) in the uninjured P65 nerve.

In summary, early postnatal SCs are characterized by the continued expression of Sox10, Sox9, Jun, Oct6 and Yy1, the acquisition of Nfatc4 and Egr2 protein expression, and the loss of AP2α expression. In contrast, late postnatal (i.e., adult) SCs initiate Sox2 and Egr1 expression at low levels, and begin to downregulate Jun and Oct6 expression.

### Nerve injury triggers adult Schwann cells to recapitulate a unique pattern of embryonic glial lineage transcription factors

Peripheral nerve injury has been suggested to induce SC de-differentiation and an iSC phenotype [11, 17]. However, while there are clearly global changes in SC gene expression post-injury [17, 40, 62], whether a specific embryonic SC stage is recapitulated, and which set of gliogenic transcription factors are deregulated, remains poorly understood. We thus assessed our panel of transcription factors in “repair” SCs by performing a crush injury on the P60 sciatic nerve, and assessing gene expression at P65, 5 days post injury (dpi).

**Fig 9:**
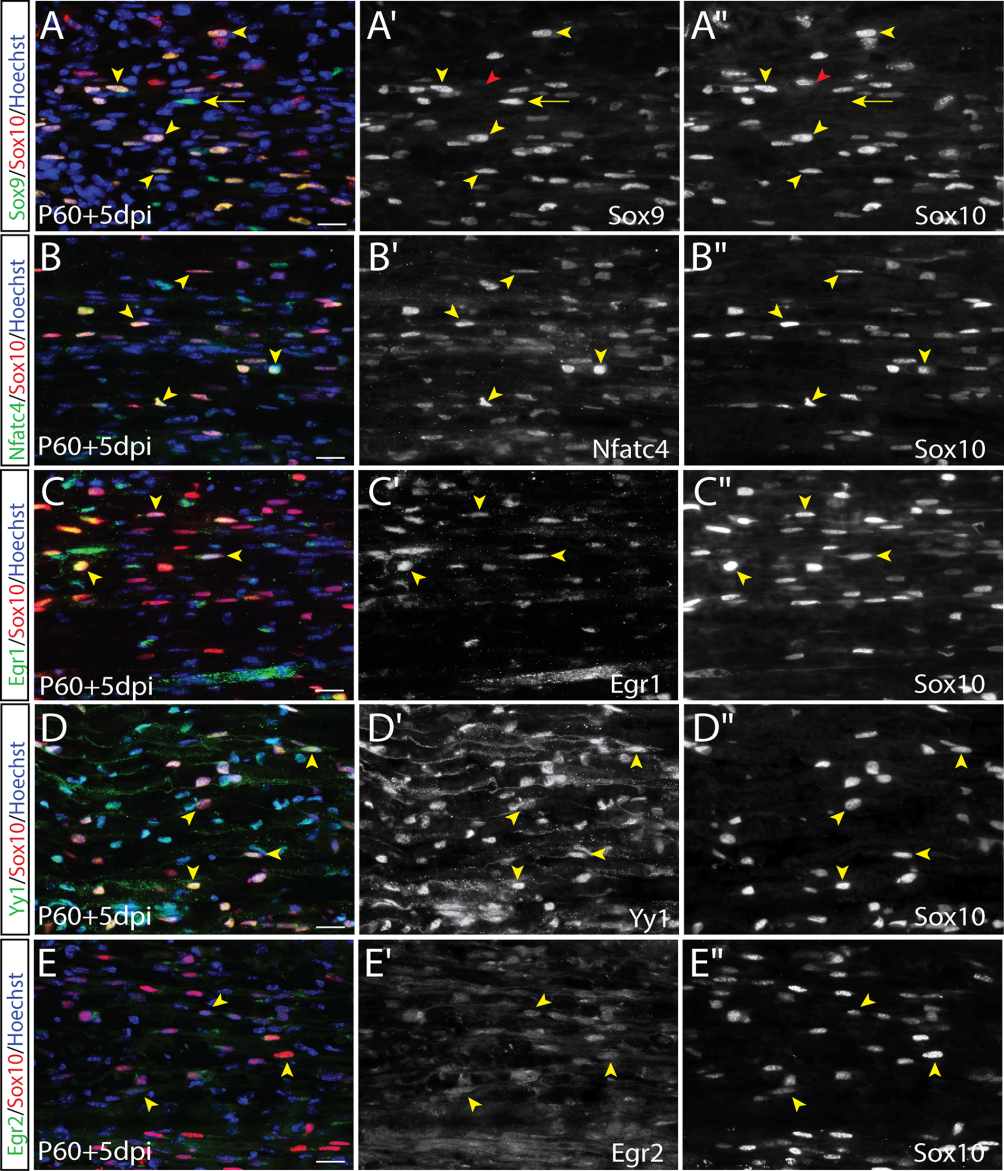
Expression of SC lineage markers in P65 sciatic nerve after acute nerve injury. (A-E) Co-labeling of Sox10 (red) with Sox9 (A-A''), Nfatc4 (B-B''), Egr1 (C-C''), Yy1 (D-D''), and Egr2 (E-E'') in longitudinal sections of the injured P65 sciatic nerve. Merged images of protein of interest (green), Sox10 (red) and Hoechst (blue) (A-E). Yellow arrowheads indicate co-labelled cells. Scale bars, 20μm.

**Fig 10:**
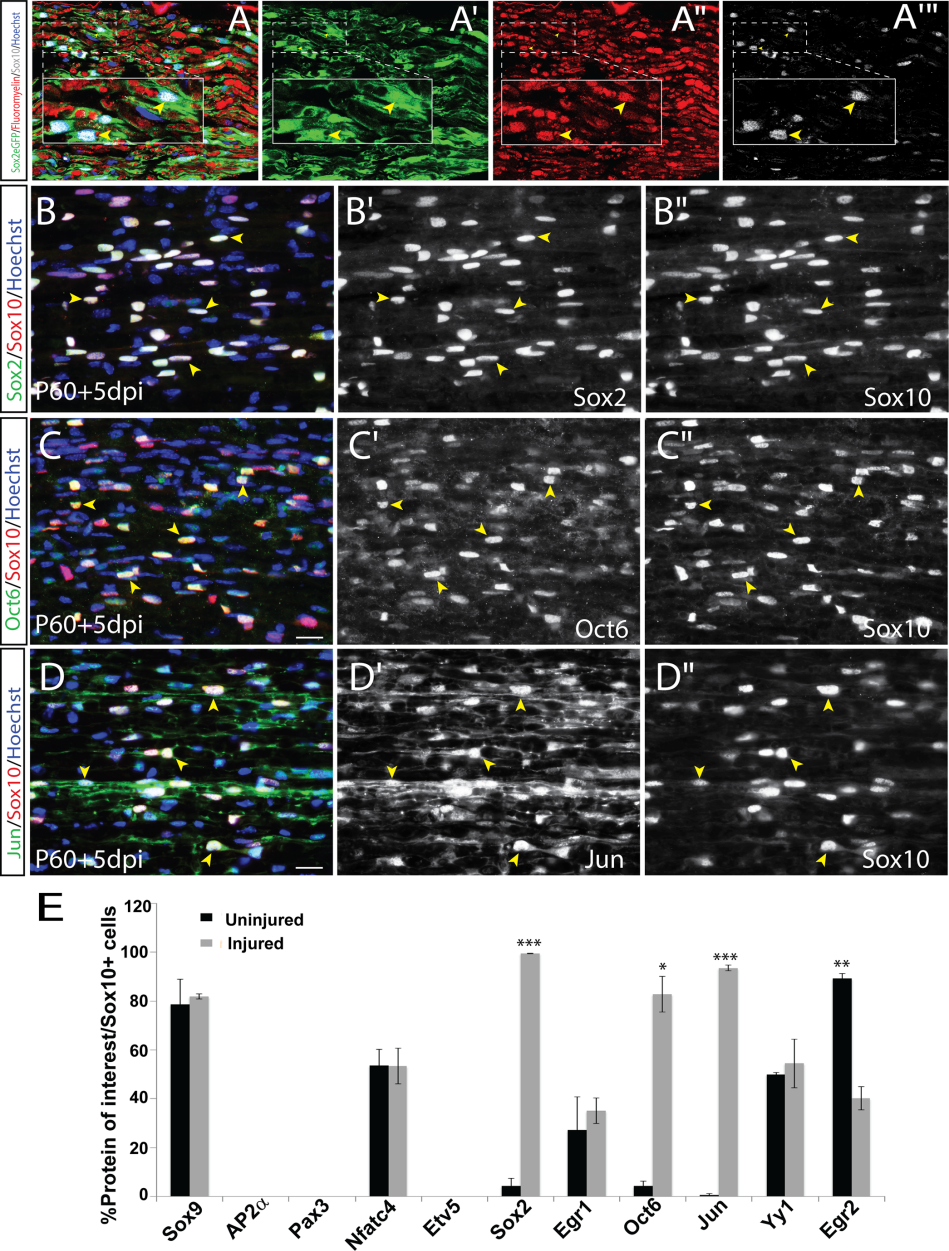
Developmental glial-lineage genes are up-regulated after acute nerve injury. (A-F) Co-labeling of Sox10 (grey) with fluoromyelin (red) and GFP (green) in the distal stump of Sox2-eGFP mice after 5 dpi (A-A‴). Co-labeling of Sox10 with Sox2 (B-B''), Oct6 (C-C''), and Jun (D-D''). Merged images of protein of interest (green), Sox10 (red) and Hoechst (blue) (A-E). Yellow arrowheads indicate co-labelled cells. Quantification of percentage of Sox10^+^ cells that co-express each of the proteins of interest (F). Error bars = S.E.M. Scale bars, 20μm.

Similar to uninjured P65 nerves, Sox9 was expressed in the majority of Sox10^+^ cells in the distal stump at 5 dpi (81.9±3.6% Sox9^+^Sox10^+^/Sox10^+^ SCs; Fig 9A–9A'' vs 88.4±0.3% in uninjured nerve; Fig 8B–8B'', Fig 10E). Singly labelled Sox9^+^ (yellow arrows) and Sox10^+^ (red arrowheads) SCs are marked to depict specificity of the antibodies (Fig 9A–9A''). In addition, the ratios of Sox10^+^ SCs expressing Nfatc4 (53.5±6.2% Nfatc4^+^Sox10^+^/Sox10^+^ SCs; Fig 9B–9B'' vs. 53.3±6.8% uninjured; Fig 8C–8C'', Fig 10E) and Yy1 (55.9±7.6% Yy1^+^Sox10^+^/Sox10^+^ SCs; Fig 9D–9D'' vs. 50.0±0.6% in uninjured nerve; Fig 8H–8H'', Fig 10E) were not significantly altered post-injury. Notably, although the frequency of Egr1^+^Sox10^+^ cells did not change after injury (35.1±5.3% Egr1^+^Sox10^+^/Sox10^+^ SCs; Fig 9C–9C'' vs 27.1±13.6% uninjured; Fig 8E–8E'', Fig 10E), the Egr1 protein was distinctively localized to the nucleus and was intensified relative to uninjured nerves (where expression was cytoplasmic), suggestive of an active role in transcription and/or altered function in denervated SCs. In contrast, the frequency of Egr2^+^Sox10^+^ cells within the distal stump was significantly diminished at 5dpi (40.17 ± 4.67% Egr2^+^Sox10^+^/Sox10^+^ SCs; Fig 9E–9E'' vs. 89.22 ± 2.1% in uninjured nerve; Fig 8I–8I'', Fig 10E). However, Egr2 expression remained in nearly 40% of Sox10+ cells, but the intensity was diminished relative to pre-injury levels, consistent with previous reports [35], which showed that Egr2 expression declined one week post-injury. Downregulation of Egr2 following compression injuries has been previously reported [63], and is expected, since Egr2 is required to initiate a terminal myelination program [35], and SCs limit myelin production post-injury to facilitate repair and enable cell cycle re-entry [22, 34, 64, 65]. Interestingly, although the frequency of Yy1^+^ Sox10^+^ and Nfatc4^+^ Sox10^+^ cells did not change after injury, many SCs also co-expressed the mitotic indicator Ki67 (S12A and S12C and S12D and S12F Fig). Because injured SCs co-express pro-myelinating/myelinating genes, as well as Ki67 and Sox2, which is expressed in nearly all SCs post injury, myelinating genes do not inhibit the proliferative capacity of de-differentiating SCs.

We next assessed the expression of transcription factors that were only rarely observed within the uninjured P65 nerve, including Sox2, Jun, and Oct6. To do this, we performed crush injuries in Sox2-eGFP mice. Distal to the injury, the majority of Sox10^+^ SCs were associated with GFP (>95%), and were also associated with degraded fluoromyelin^+^ myelin segments (Fig 10A–10A‴). Consistent with this observation, 99.5±0.3% of Sox10^+^ SCs expressed Sox2 after 5 dpi (Fig 10B–10B'', Fig 10E), reflecting a significant increase in Sox2 expression in repair SCs. Similarly, the majority of Sox10^+^ SCs distal to the injury site initiated Jun expression (93.5±1.9% Jun^+^Sox10^+^/Sox10^+^ SCs; Fig 10D–10D'', Fig 10E), which has been reported to play a pivotal role during nerve repair [40, 66]. We also detected a significant upregulation in Oct6 expression (83.7±6.1% Oct6^+^Sox10^+^/Sox10^+^ SCs; Fig 10C–10C'', Fig 10E). Notably, while Sox2 is expressed in E12.5 SCPs, during embryonic development, Jun and Oct6 are not expressed until E18.5 in late immature/pro-myelinating SCs, indicating that the repair phenotype does not faithfully recapitulate all characteristics of either embryonic stage.

We failed to detect Pax3 protein in Sox10^+^ SCs 5 dpi (S11J–S11L Fig), even though *Pax3* transcripts have been isolated from SCs following acute injury [43]. Similarly, Etv5, which marks satellite glial cells and is downregulated in maturing SCs [42], was not expressed in Sox10^+^ SCs (S11P–S11R Fig). Finally, AP2α, which is expressed in SCPs and involved in negatively regulating SC maturation [39], was also absent in the distal segment at 5 dpi (S11D-F).

In summary, with the exception of Egr2, SCs retain the expression of the core transcriptional program of a SC identity post-injury, including Sox9, Nfatc4 and Yy1. In addition, a ‘repair’ SC phenotype is characterized by an increase in expression of the SC lineage markers Sox2, Jun and Oct6, but other embryonic SC markers (AP2α, Pax3 and Etv5) are not induced, suggesting that SCs do not de-differentiate to a particular embryonic state.

## Discussion

**Fig 11:**
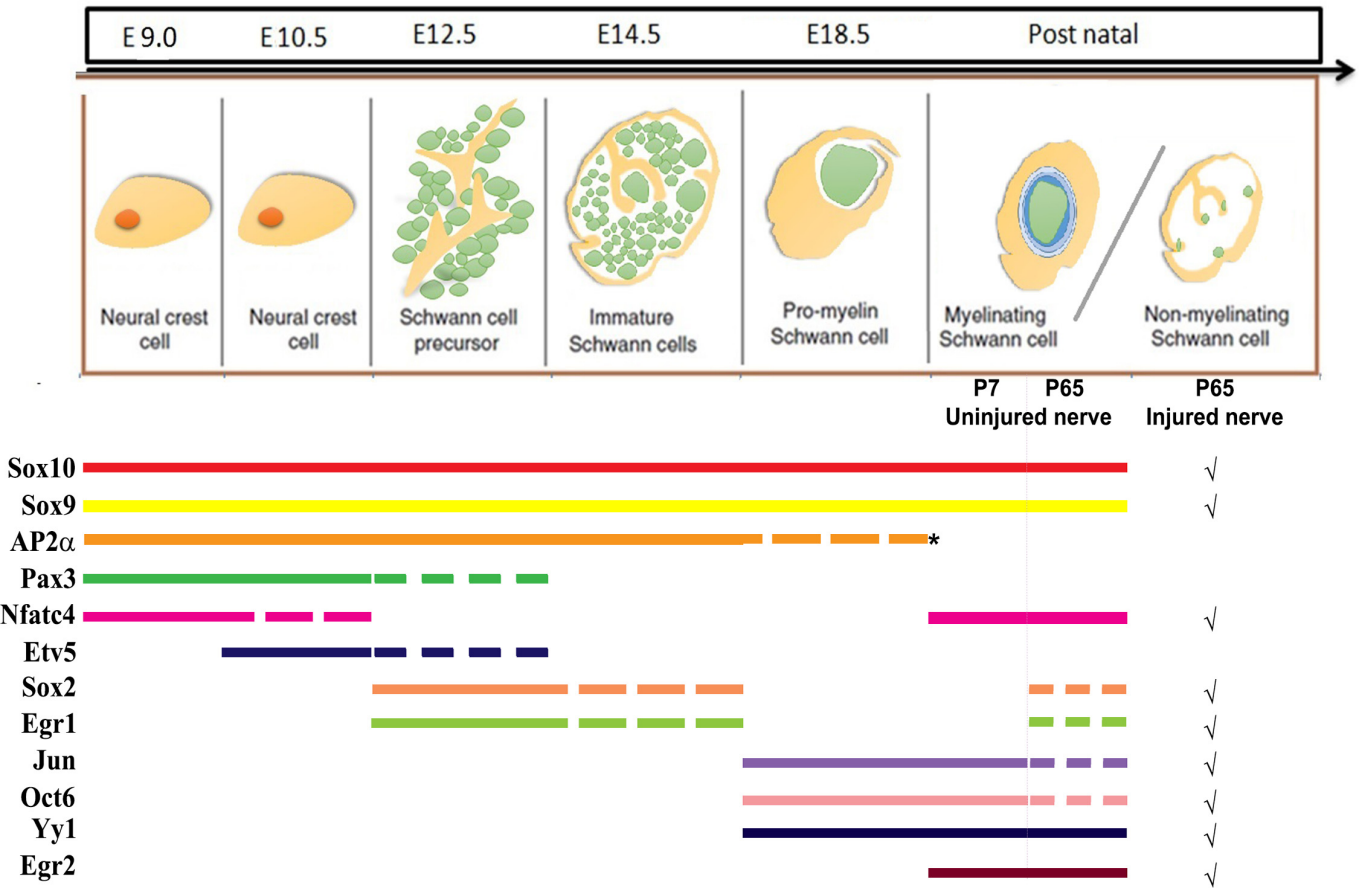
Summary of temporal expression profiles of key transcription factors in the SC lineage. Sox9 and Sox10 are expressed throughout SC genesis, beginning at E9.0 in migrating NCCs and persisting until P65 in myelinating and non-myelinating SCs in the sciatic nerve. AP2α, Pax3 and Etv5 are also expressed in NCCs, persisting until E12.5 in SCPs with Nfatc4 expression being restricted to NCCs. Egr1 and Sox2 expression is initiated in SCPs at E12.5, persisting until E14.5 in iSCs. At E18.5, AP2α is expressed in the DRG but is undetectable in the nerves while the expression of Jun, Oct6, and Yy1 is initiated and persists till P65. Rare Sox2^+^Sox10^+^ positive cells are also detected in the P65 nerve. Egr2 expression is not detected until P7 in myelinating SCs in the sciatic nerve. Post-injury, high upregulation in expression of Sox2, Oct6, and Jun is observed, while distinct nuclear Egr1 expression is also detected. The straight lines represent continued expression of the markers through the different stages, while the dotted lines represent declining or low expression. The asterisk indicates where expression is restricted to the DRG and is undetectable in the nerves at E18.5. Markers expressed in the nerve after an acute injury have been denoted with ‘√’. The green cells represent the developing axon, while the beige cells represent the NCCs at E9.0-E10.5 and the developing SCs at E12.5 to post-natal stages.

Generation of SCs from NCCs is a progressive process characterized by at least five transient embryonic stages of development. Here we have defined these developmental stages by examining the expression patterns of 12 transcription factors (Sox2, Sox9, Sox10, AP2α, Pax3, Nfatc4, Etv5, Jun, Yy1, Egr1, Egr2, Oct6) (Fig 11). While other studies have performed more global analyses of gene expression in SC lineages between E9.5 and P0 [67], similarly delineating the dynamic changes that occur over these time points, our focus on protein expression levels of a core set of transcription factors provides a framework for scientists to reliably follow temporal identity transitions in this lineage. Based on this temporal profile, we can begin to delineate the potential diverse functional contributions of these genes during SC development and their recapitulation following peripheral nerve injury.

At E9.0, the earliest NCC stage is characterized by the expression of Sox10, Sox9, AP2α, Pax3 and Nfatc4, a gene expression profile that is maintained at E10.5. However, a distinct feature of NCC precursors coalescing in the DRG is the initiation of Etv5 expression and concomitant loss of Nfatc4 expression. Next, E12.5 SCPs in the DRG and dorsal and ventral roots retain the expression of Sox10, Sox9, AP2α, and Pax3 while also initiating the expression of Sox2 and Egr1, but losing the expression of Etv5. This is followed at E14.5 by the loss of Pax3 expression, while Sox10, Sox9, AP2α, Sox2 and Egr1 continue to be expressed in iSCs. A more dramatic change in gene expression is observed in E18.5 late immature/pro-myelinating SCs, which express Sox10, Sox9, Jun, Oct6 and Yy1, but lose the expression of AP2α, Sox2 and Egr1 in the nerve. At P7 and P65, we observed a very similar expression profile as seen at E18.5 (i.e., Sox10, Sox9, Jun, Oct6, Yy1) with the added expression of Nfatc4 and Egr2 at P7 and P65, and Egr1 and Sox2 expression in the P65 nerve.

Following peripheral nerve injury, SCs assume a transient ‘de-differentiated’ phenotype that is critical for supporting axonal regeneration and nerve repair. Hence, we characterized expression of the transcription factor panel following injury in the adult P65 nerve, comparing it against the embryonic and postnatal profile. We reveal that denervated SCs upregulate the expression of only a subset of early glial-lineage transcription factors. Indeed, expression of genes associated with both SCPs (Sox2, Egr1) and pro-myelinating/late immature SCs (Oct6, Jun) are elevated following denervation of adult SCs, while genes involved in the myelination program are actively lost (Egr2). Importantly, the absence of expression of several embryonic genes in denervated SCs (AP2α, Etv5, Pax3) may provide a strategy to enhance or prolong the repair phenotype to improve recovery of function following PNS injury or neuropathy.

### Sustained expression of Sox9 and Sox10 across the Schwann cell lineage

An intricate network of transcription factors controls the timely development of peripheral glia, including several of the transcription factors examined in this study. One of the core regulators of SC development is Sox10, which we used to mark peripheral glial cells in our co-labeling experiments. Indeed, we (this study) and others [26, 28] found that Sox10 is continually expressed in Schwann and satellite glial cells throughout development (Figs 1–9) and into adulthood. Prior studies have revealed that Sox10 is required for the specification and terminal differentiation of iSCs, and to maintain a peripheral glial phenotype [28, 49]. Mechanistically, Sox10 directly activates Egr2 expression, acting synergistically with Oct6 and Nfatc4 [30–34] to induce the expression of peripheral myelin genes such as myelin basic protein (MBP), myelin protein zero (MPZ), myelin associated glycoproteins and connexin-32 (Cx32) [32, 34, 45]. Consequently, deletion of Sox10 results in loss of Egr2 expression, as well as myelin sheath degeneration and axonal death resulting in declined nerve conduction [29]. However, conditional Sox10 ablation studies also revealed that Sox10 is essential for survival of early migrating trunk NCCs, but not the survival of adult SCs, indicating that it is a critical player early in the SC lineage [29, 68, 69] and later to maintain functional myelination.

Interestingly, we also found that Sox9 follows a similar temporal pattern with sustained, overlapping expression with Sox10. Sox9 induces a NCC phenotype [25], and its expression biases migrating NCCs towards glial and melanocyte lineage selection [26]. While previous studies have observed Sox9 expression in the peripheral nerve at E14.5 [58], earlier stages of Sox9 expression have not been documented. Interestingly, recent work has suggested that isolated human SCs show negligible Sox9 expression, but Sox9 is over-expressed in neurofibromatosis 1 tumor derived SCs [70], hinting to a role for Sox9 in promoting SC proliferation. It is possible that sustained Sox9 activation in Schwann cells may enable re-establishment of the immature SC phenotype and re-entry into the cell cycle, and should be addressed in future studies.

### Transcriptional regulators expressed at early stages in the Schwann cell lineage

In addition to Sox9 and Sox10, four other transcriptional regulators in our panel were expressed at the NCC stage; AP2α, Pax3, and Etv5, with Etv5 appearing one day later than the others. One novel observation was that Nfatc4, which is a calcium-responsive transcription factor, exhibits transient early expression in NCCs but is rapidly lost by E10.5, before re-initiating expression in maturing SCs at P7. A role for Nfatc4 in early NCCs has not previously been reported, however at later stages, Nfatc4 has been reported to bind a myelin specific enhancer in Egr2, cooperatively with Sox10, to activate Egr2 and other myelin genes during the pro-myelination to myelination transition [45].

AP2α expression persists until 18.5, however, AP2α^+^Sox10^+^ cells at this stage are confined to the DRG, and AP2α expression is not seen in the Sox10^+^ SCs lining the nerve. This could be suggestive of a role for AP2α in satellite glial cells in the DRG. Also, AP2α is co-expressed with majority of Sox10^+^ cells throughout development, except at E18.5 when AP2α expression becomes restricted to the DRG. Interestingly, overexpression of AP2α *in vitro* blocks the transition of SCPs to iSCs [39], even though we found that this transcription factor is expressed in iSCs and pro-myelinating SCs. The *in vivo* requirement for AP2α in the SC lineage has not yet been determined.

Pax3 is co-expressed with most Sox10^+^ glial lineage cells at early embryonic stages, and may play a role in regulating the proliferation of these early glial cells. Indeed, *Pax3* induces proliferation in SCPs, and *Pax3* transcript levels decline at the onset of differentiation [43]. Finally, we also found that Etv5 expression is limited to E10.5 NCC precursors in the DRG and in satellite glia cells at later stages. The function of Etv5 in the SC lineage has not been elucidated, although misexpression of dominant negative Etv5 in NCCs affects neuronal and not glial specification [54]. The absence of Etv5 at all later time points of the SC lineage (including after injury) suggest that it does not play a role in SC differentiation or myelination.

### Transcriptional regulators expressed at late stages in the Schwann cell lineage

Several of the transcription factors in our panel were not expressed in NCCs, but were expressed in definitive cells in the SC lineage, including Sox2, Egr1, Jun, Oct6, Yy1 and Egr2. In contrast to Sox9 and Sox10, Sox2 is expressed in a more limited window of SC development, appearing in E12.5 SCPs and E14.5 iSCs. A decline in number of Sox2^+^Sox10^+^ cells is observed at E14.5. This data is consistent with previous studies demonstrating that Sox2 expression declines upon neuronal commitment, and continues at low levels in SCPs and iSCs [23]. Interestingly, persistent Sox2 expression suppresses myelin-associated genes such as Egr2 and MPZ whilst maintaining cells in an undifferentiated state [22]. Notably, cross-repressive interactions between Sox2 and Mitf/Egr2 regulate the differentiation of SCPs into either myelinating SCs or melanocytes [24]. Sox2 is thus considered a negative regulator of myelination.

The zinc finger transcription factors *Egr1* (Early growth response 1) and *Egr2* have nearly identical DNA binding domains but opposite effects on myelination; Egr2 (Krox-20) promotes the differentiation of SCs to a myelinating phenotype while Egr1 is a non-myelinating SC marker [16, 35]. Hence, we (this study) and others [35] found that Egr1 is expressed in SCPs but is downregulated as the cells mature. At postnatal stages, Egr1 expression is also re-initiated in non-myelinating SCs [35], where a modest cytoplasmic expression pattern is observed, and is sustained in a subset of SCs within the adult (P65) sciatic nerve. Conversely, *Egr2* transcripts are detected in the dorsal and ventral roots from E10.5 onwards but are absent from the SCs in the DRG and peripheral nerves throughout embryogenesis [15]. Strikingly, we did not detect Egr2 protein in the dorsal and ventral roots, suggesting that it may not be translated until postnatal stages. Indeed, we (this study) and others [35] observed Egr2 protein in SCs lining the postnatal peripheral nerve, which is expected considering its requirement for myelination.

We observed Jun expression in late immature/pro-myelinating SCs, P7 and in rare Jun^+^Sox10^+^ SCs in the adult nerve (P65). Downregulation of Jun expression is mediated by Egr2 just prior to onset of myelination [40]. Overexpression of Jun has been associated with a decline in myelination and de-differentiation of SCs, along with a reduction in Egr2 and MPZ levels [40]. Similarly, we detected Oct6 expression in a subset of Sox10^+^ pro-myelinating SCs at E18.5, in mature SCs at P7 and in rare cells at P65. Oct6 acts in synergy with Sox10 to induce Egr2 expression, which in turn promotes the expression of several myelin proteins [34, 37]. Oct6 deficient mice show a transient arrest at the pro-myelinating stage, which is overcome by P10, with a late onset of Egr2 expression and myelin formation [37]. Oct6 not only promotes myelination by promoting terminal differentiation from pro-myelinating to myelinating SC via induction of Egr2, but also prevents premature myelination by repression of MBP and MPZ. A progressive reduction in Oct6 levels allows MBP and MPZ to be activated, thereby initiating a temporally controlled myelination program. Notably, constitutive overexpression of Oct6 results in a persistent hypomyelination phenotype in mice and gradual axonal loss [71], suggesting that varied levels of Oct6 allow for diverse functional contributions across the SC lineage.

Yy1, expressed at E18.5 and postnatally at P7/P65, is important for attaining the myelination phenotype, such that conditional knockdown of Yy1 in SCs results in hypomyelinated nerves with deficient expression of MPZ and Pmp22 [36]. The Egr2 promoter and Myelinating Schwann cell Element (MSE) has multiple binding sites for Yy1. Activation of MEK pathway occurs in response to the axonal signaling molecule, Neuregulin1. This in turn results in serine phosphorylation of Yy1. The phosphorylated Yy1 is recruited to binding sites in the Egr2 promoter and MSE, activating Egr2 expression [36] and underscoring its important role in SC myelination.

### Injury activates SC lineage genes that recapitulate features of both Schwann cell precursors and pro-myelinating Schwann cells

Following a nerve crush injury we observed continued expression of Sox10 in nerve SCs and co-expression with Sox9, Nfatc4 and Yy1. Previous work showed that Sox9 is expressed in isolated SCs from P3 nerves [58] but its presence in adult SCs *in vivo* or following injury has not been reported. Constitutive expression of Sox9 in adult (uninjured) SCs and following injury *in vivo*, suggesting that Sox9 may play a continued role in the maintenance of the SC fate or in sustaining SC competence to re-acquire a de-differentiated state, particularly since it has a demonstrated role in both induction and maintenance of self-renewal capacity in subependymal neural stem cells [72] and various epithelial stem cell types [73, 74]. Indeed, SC de-differentiation encompasses the hallmark features of a stem cell, exhibiting both the capacity for self-renewal, and the ability to generate mature cell types. Future conditional knockout studies will need to be done to determine the role of Sox9 in adult SCs and its potential contribution to the de-differentiation process.

Acutely injured SCs exhibit robust activation of the myelin-inhibitory gene Sox2, as has been previously reported [22], and elevated levels of Egr1, both of which are unique to SCPs and iSCs and absent in late immature/pro-myelinating SCs (Fig 10). However, several other transcription factors that are upregulated in denervated SCs are markers of late immature/pro-myelinating SCs, including Jun, and Oct6 (Fig 11). SC de-differentiation was also associated with a concomitant loss of mature myelinating genes such as Egr2.

Egr1 is a transcriptional activator that is normally active during cell cycle-re-entry [75]. Although the frequency of Egr1^+^Sox10^+^ SCs did not change, Egr1 protein exhibited a marked increase in intensity and nuclear translocation following injury, suggesting that denervation causes a change in Egr1 function [35]. Egr1 may thus be an important modulator of SC plasticity. Egr1 and Egr2 appear to play opposing roles in modulating the acquisition of non-myelinating versus myelinating phenotypes [35]. Sustained Egr1 expression in a subset of SCs at all postnatal stages, and its known role in regulating cell cycle entry, suggests that this factor might also enable SC proliferation after injury and/or activation of other genes that are necessary for de-differentiation, partly through its known cooperation with Egr3 [76]. Future studies using conditional knockout approaches will need to be done in order to determine the ultimate role for Egr1 and Egr3 in acquisition of the de-differentiated SC state.

Interestingly, the frequency of pro-myelinating genes Yy1 and Nfatc4, did not change following injury further indicating the retention of late immature/pro-myelinating SC traits. Despite their putative pro-myelination function, many Yy1^+^ and Nfatc4^+^ cells that co-localized with Sox10, were also mitotically active, suggesting that both of these transcription factors are permissive of the proliferative, de-differentiated state.

The most notable change after injury was observed with respect to Jun and Oct6 expression. A robust increase in Jun expression has been reported in denervated SCs [40, 62]. Indeed, Jun is a critical regulator of de-differentiation such that loss of Jun results in an inability to downregulate myelination genes and severely impairs regeneration. The POU domain transcription factor Oct6 initiates the transition from pro-myelinating to myelinating SC [77]. Interestingly, a shift from cytoplasmic to nuclear localization in Oct6 expression has been reported in axonal regeneration in peripheral neuropathic conditions [78]. It is noteworthy that Oct6 expression is highly upregulated at a time when myelin is being degraded and SCs are repressing their myelin program. Oct6 could be playing multiple roles in governing SC function, a possibility that requires further exploration. Since neither Jun nor Oct6 are expressed in SCPs but only by late immature/pro-myelinating SCs at E18.5, it suggests that at the level of protein expression, the de-differentiated SC state cannot be equated to a single embryonic SC stage, but rather is unique and includes features from multiple developmental stages.

Several key transcription factors including AP2α, Etv5, and Pax3 that are associated with early stages of development were not expressed within denervated SCs. We observed only extremely rare Pax3^+^ cells in the adult nerve (data not shown). A recent report suggests that Pax3 labels approximately 1% of cells in the adult nerve and marks non-myelinating SCs [44]. This suggested that possibly only a subset of Sox10^+^ non-myelinating SCs express Pax3. Notably, we did not observe Pax3 expression following injury either, despite seeing robust expression in NCCs that were immunostained in parallel as a positive control. This is in contrast to a previous study [43] that reported *Pax3* transcripts were detected in the denervated distal stump at seven days post transection injury. This discrepancy may be due to several factors: 1) the temporal expression profile of Pax3 may be delayed, such that it does not peak until after the 5 day time-point we examined, 2) severity of nerve injury (transection versus crush) may be an important determinant of the SC transcriptional response within denervated SCs, 3) young mice (3 weeks of age) may elicit a different response compared to the adult animals (P65) used in our study, and 4) *Pax3* transcripts may not be translated. Future studies using a conditional Pax3 gene deletion could determine its role in establishing the SC repair phenotype after injury.

Our spatio-temporal expression study provides a comprehensive glimpse into the expression profiles of the various transcriptional regulators involved in SC development and in SC injury response. To summarize, the injured peripheral nerve contains a highly dynamic and heterogeneous population of glia that undergoes phenotypic reversion to a de-differentiated state by recapitulating a subset of early glial-associated transcription factors. Taken together, we provide evidence that “repair” SCs retain their core SC transcriptional program, while initiating the expression of a subset of embryonic genes that represent several embryonic SC stages. Since SC function is diminished in the adult aging PNS it may be necessary to artificially activate additional genes, particularly those that fail to be re-activated or are diminished following prolonged denervation, in order to maximize nerve regeneration.

## Supporting Information

S1 Fig Expression of SC lineage markers in E9.0 NCCs. (A-U) Co-expression of Etv5 (A-C), Jun (D-F), Sox2 (J-L), Yy1 (M-O), Egr1 (P-R), and Egr2 (S-U) with Sox10, and co-expression of Oct6 with Sox9 (G-I) in transverse sections through the E9.0 trunk. A,D,G,J,M,P,S are merged images of the protein of interest in red and Sox10 (or Sox9) in green. Blue is DAPI counterstain. B,E,H,K,N,Q,T show expression profiles of the protein of interest, while C,F,I,L,O,R,U shows Sox10 (or Sox9) expression. Green asterisk indicates background staining from red blood cells. nt, neural tube. Scale bars, 40μm.
(TIF)

S2 Fig Co-labeling of SC lineage markers and NeuN in the E10.5 trunk. (A-L) Co-labeling of NeuN and Sox9 (A-C), Sox10 (D-F), AP2α (G-I), and Etv5 (J-L) in transverse sections through the E10.5 trunk. Low magnification merged images of the protein of interest (red) with NeuN (green) (A,D,G,J). Blue is DAPI counterstain. High magnification images of the DRG, showing merged images of the protein of interest (red) and NeuN (green) (B,E,H,K), and single protein of interest images in white (C,F,I,L). drg, dorsal root ganglion; nt, neural tube; scg, sympathetic chain ganglion; sn, spinal nerve; vr, ventral root. Scale bars, 60μm.
(TIF)

S3 Fig Expression of Sox9 and Sox10 in wild type cortices. (A-I) Co-labeling of Sox10 with Sox9 in sagittal sections of wild type E13.5 (A-C), E15.5 (D-F), and E18.5 (G-I) cortices. Merged images of Sox9 in red and Sox10 in green (A,D,G). Blue is DAPI counterstain. Expression profiles of Sox9 (B,E,H) and Sox10 (C,F,I). Red arrows indicate co-expression of Sox9 with Sox10, while green arrows mark Sox9+Sox10- cells. Scale bars, 60μm.
(TIF)

S4 Fig Expression of SC lineage markers in E10.5 NCC precursors. (A-DD) Co-labeling of Sox9 with Oct6 (A-E), and Sox10 with Jun (F-J), Sox2 (K-O), Yy1 (P-T), Egr1 (U-Y), and Egr2 (Z-DD) in transverse sections through the E10.5 trunk. Merged images of the protein of interest (red) with Sox10 or Sox9 (green) (A,F,K,P,U,Z). Blue is DAPI counterstain. High magnification images of the DRG, showing merged images of the protein of interest (red) and Sox10 (green) (B,G,L,Q,V,AA), and single protein of interest images in white (C,H,M,R,W,BB). High magnification images of the ventral roots, showing merged images of the protein of interest (red) and Sox10 (green) (D,I,N,S,X,CC), and single protein of interest images in white (E,J,O,T,Y,DD). drg, dorsal root ganglion; nt, neural tube; scg, sympathetic chain ganglion; sn, spinal nerve; vr, ventral root. Scale bars, 60μm.
(TIF)

S5 Fig Co-labeling of SC lineage markers and NeuN in the E12.5 trunk. (A-U) Co-labeling of NeuN with Sox9 (A-C), Sox10 (D-F), AP2α (G-I), Sox2 (J-L), Etv5 (M-O), Jun (P-R), Egr1 (S-U). Low magnification merged images of the protein of interest (red) with NeuN (green) (A,D,G,J,M,P,S). Blue is DAPI counterstain. High magnification images of the DRG, showing merged images of the protein of interest (red) and NeuN (green) (B,E,H,K,N,Q,T), and single protein of interest images in white (C,F,I,L,O,R,U). Arrows indicate co-expression of proteins of interest with NeuN in DRG sensory neurons. drg, dorsal root ganglion; sc, spinal cord. Scale bars, 60μm.
(TIF)

S6 Fig Expression of SC lineage markers in E12.5 SCPs. (A-II) Co-expression of Sox9 with Oct6 (H-N), and Sox10 co-expression with Nfatc4 (A-G), Jun (O-U), Yy1 (V-BB), and Egr2 (CC-II) in transverse sections through the E12.5 trunk. Low magnification merged images of protein of interest (red) and Sox9 (green, H) or Sox10 (A,O,V,CC green). Blue is DAPI counterstain. High magnification images of the DRG, showing merged images of the protein of interest (red) and Sox9 (I; green) or Sox10 (B,P,W,DD; green), and single protein of interest images in white (C,J,Q,X,EE). High magnification images of the dorsal roots, showing merged images of the protein of interest (red) and Sox9 (K; green) or Sox10 (D,R,Y,FF; green), and single protein of interest images in white (E,L,S,Z,GG). High magnification images of the ventral roots, showing merged images of the protein of interest (red) and Sox9 (M; green) or Sox10 (F,T,AA,HH; green), and single protein of interest images in white (H,N,U,BB,II). dr, dorsal root; drg, dorsal root ganglion; sc, spinal cord; scg, sympathetic chain ganglion; sn, spinal nerve; vr, ventral root. Scale bars, 60μm.
(TIF)

S7 Fig Expression of SC lineage markers in in E14.5 iSCs. (A-WW) Co-expression of Sox10 with Pax3 (A-G), Nfatc4 (H-N), Etv5 (O-U) Jun (V-BB), Yy1 (JJ-PP), Egr2 (QQ-WW), and Sox9 with Oct6 (CC-II) in transverse sections through the E14.5 trunk. Low magnification merged images of protein of interest (red) and Sox10 (A,H,O,V,JJ,QQ; green) or Sox9 (CC; green). Blue is DAPI counterstain. High magnification images of the DRG, showing merged images of the protein of interest (red) and Sox10 (B,I,P,W,KK,RR; green) or Sox9 (DD; green), and single protein of interest images in white (C,J,Q,X,EE,LL,SS). High magnification images of the dorsal roots, showing merged images of the protein of interest (red) and Sox10 (D,K,R,Y,MM,TT; green) or Sox9 (FF; green), and single protein of interest images in white (E,L,S,Z,GG,NN,UU). High magnification images of the ventral roots, showing merged images of the protein of interest (red) and Sox10 (F,M,T,AA,OO,VV; green) or Sox9 (HH; green), and single protein of interest images in white (G,N,U,BB,II,PP,WW). dr, dorsal root; drg, dorsal root ganglion; sc, spinal cord; vr, ventral root. Scale bars, 60μm.
(TIF)

S8 Fig Expression of SC lineage markers in E18.5 late immature/pro-myelinating SCs. (A-R) Co-labeling of Sox10 with Pax3 (A-C), Etv5 (D-F), Sox2 (G-I), Nfatc4 (J-L), Egr1 (M-O) and Egr2 (P-R). Low magnification merged images of protein of interest (red) and Sox10 (green) (A,D,G,J,M,P). High magnification images of the DRG, showing merged images of the protein of interest (red) and Sox10 (green) (B,E,H,K,N,Q), and single protein of interest images in white (C,F,I,L,O,R). Blue is DAPI counterstain. drg, dorsal root ganglion; sc, spinal cord; sn, spinal nerve; vr, ventral root. Scale bars, 60μm.
(TIF)

S9 Fig Expression of SC lineage markers in SCs. (A-V) Co-labeling of Sox10 with Egr2 (Bioss Antibodies) (A-E & F-H), Egr2 (Abcam) (I-M & N-P) in transverse sections of the E12.5 trunk (A,I) and longitudinal sections of post-natal sciatic nerve (F,N). Merged images of the protein of interest in red and Sox10 in green (A,F,I,N). Blue is DAPI counterstain. RNA *in situ* hybridisation analysis of *Egr2* in transverse trunk sections at E12.5 (Q-S) and E18.5 (T-V). Scale bars, 60μm.
(TIF)

S10 Fig Expression of SC lineage markers in P7 myelinating/non-myelinating SCs. (A-O) Co-labeling of Sox10 with AP2α (A-C), Pax3 (D-F), Etv5 (G-I), Sox2 (J-L), and Egr1 (M-O) in longitudinal sections of the P7 sciatic nerve. Merged images of the protein of interest in red and Sox10 in green (A,D,G,J,M). Blue is DAPI counterstain. Expression profiles of the protein of interest (B,E,H,K,N) and Sox10 (C,F,I,L,O). Scale bars, 40μm.
(TIF)

S11 Fig Expression of SC lineage markers absent in the P65 adult uninjured and injured nerve. (A-R) Co-labeling of Sox10 with AP2α (A-C & D-F), Pax3 (G-I & J-L), and Etv5 (M-O & P-R). Merged images of protein of interest (green), Sox10 (red) and Hoechst (blue) (A,D,G,J,M,P). Scale bars, 20μm.
(TIF)

S12 Fig Expression of pro-myelinating genes Yy1 and Nfatc4 is sustained in proliferating de-differentiated SCs. Images showing de-differentiated Schwann cells expressing the promyelinating genes (A-C) Nfatc4 (red) and (D-F) YY1 (red) are mitotically active as indicated by co-localization with Ki67 (green; arrows). Nuclei are stained with Hoechst (blue). Scale bars, 20μm.
(TIF)
