# Supplementary figures and images for: Temporal Analysis of Gene Expression in the Murine Schwann Cell Lineage and the Acutely Injured Postnatal Nerve
Source: PLoS One. 2016 Apr 8;11(4):e0153256. doi: 10.1371/journal.pone.0153256 (PMC4826002; doi:10.1371/journal.pone.0153256)

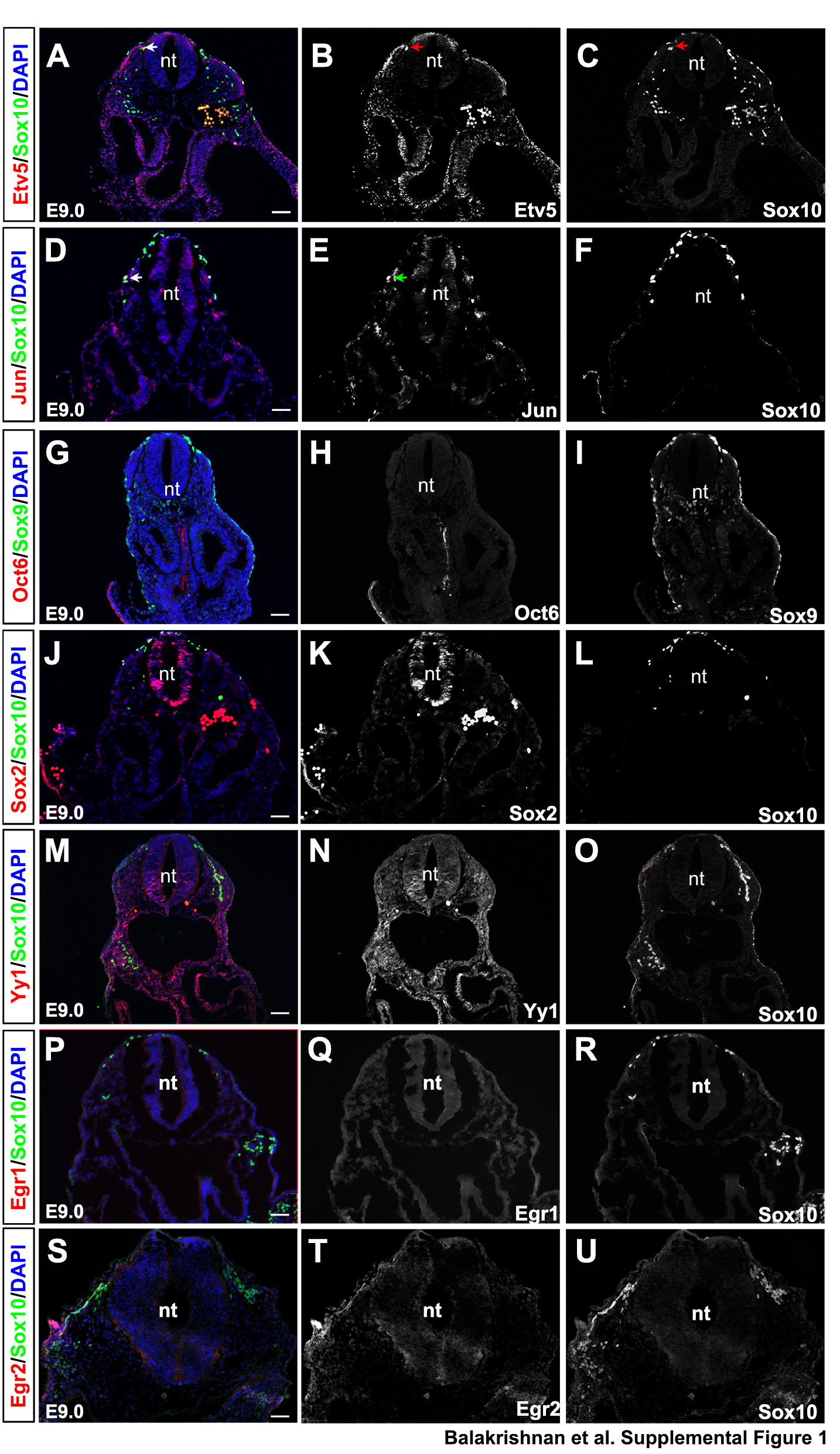

Supplement: S1 Fig — (A-U) Co-expression of Etv5 (A-C), Jun (D-F), Sox2 (J-L), Yy1 (M-O), Egr1 (P-R), and Egr2 (S-U) with Sox10, and co-expression of Oct6 with Sox9 (G-I) in transverse sections through the E9.0 trunk. A,D,G,J,M,P,S are merged images of the protein of interest in red and Sox10 (or Sox9) in green. Blue is DAPI counterstain. B,E,H,K,N,Q,T show expression profiles of the protein of interest, while C,F,I,L,O,R,U shows Sox10 (or Sox9) expression. Green asterisk indicates background staining from red blood cells. nt, neural tube. Scale bars, 40μm. (TIF) [file pone.0153256.s001.tif]

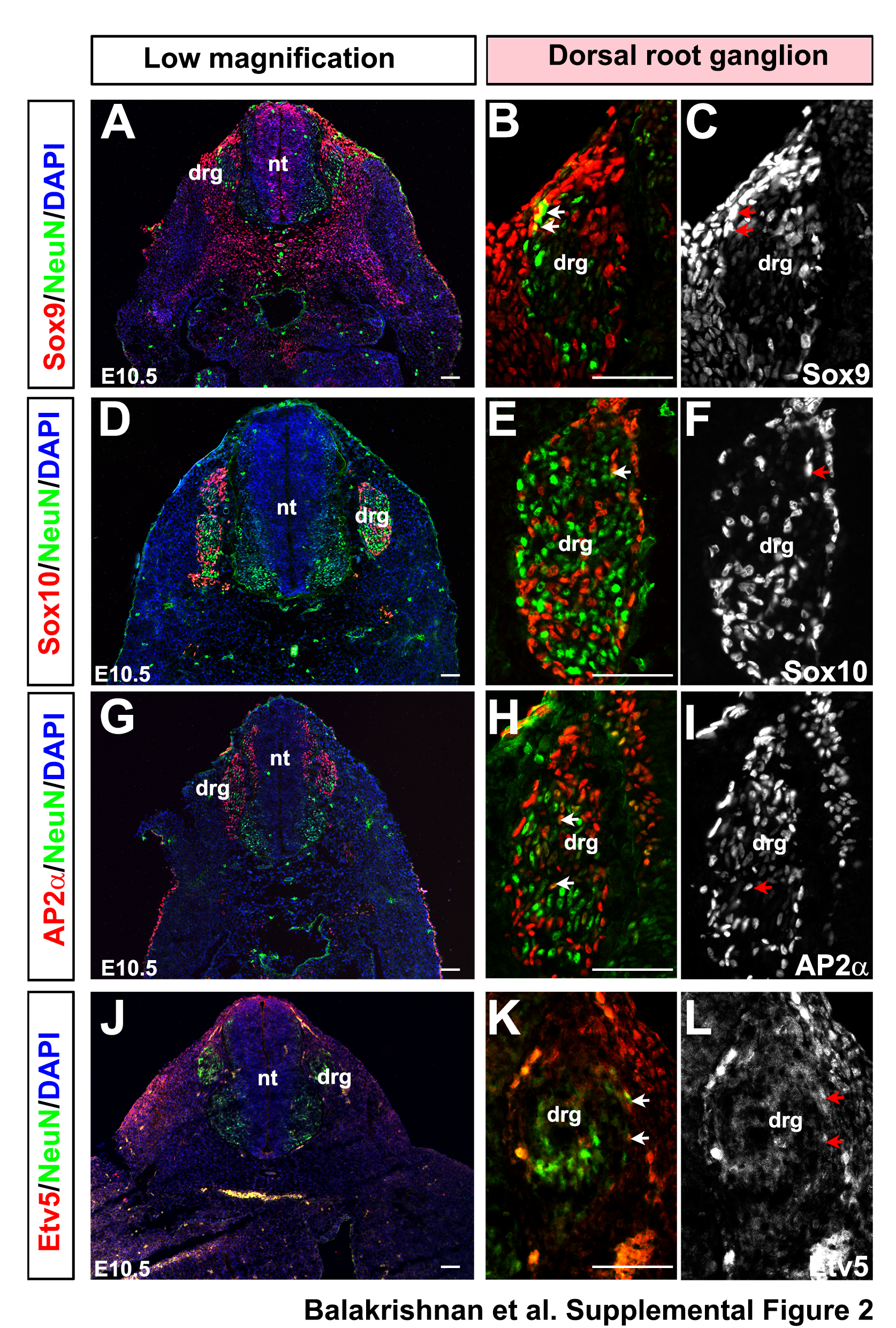

Supplement: S2 Fig — (A-L) Co-labeling of NeuN and Sox9 (A-C), Sox10 (D-F), AP2α (G-I), and Etv5 (J-L) in transverse sections through the E10.5 trunk. Low magnification merged images of the protein of interest (red) with NeuN (green) (A,D,G,J). Blue is DAPI counterstain. High magnification images of the DRG, showing merged images of the protein of interest (red) and NeuN (green) (B,E,H,K), and single protein of interest images in white (C,F,I,L). drg, dorsal root ganglion; nt, neural tube; scg, sympathetic chain ganglion; sn, spinal nerve; vr, ventral root. Scale bars, 60μm. (TIF) [file pone.0153256.s002.tif]

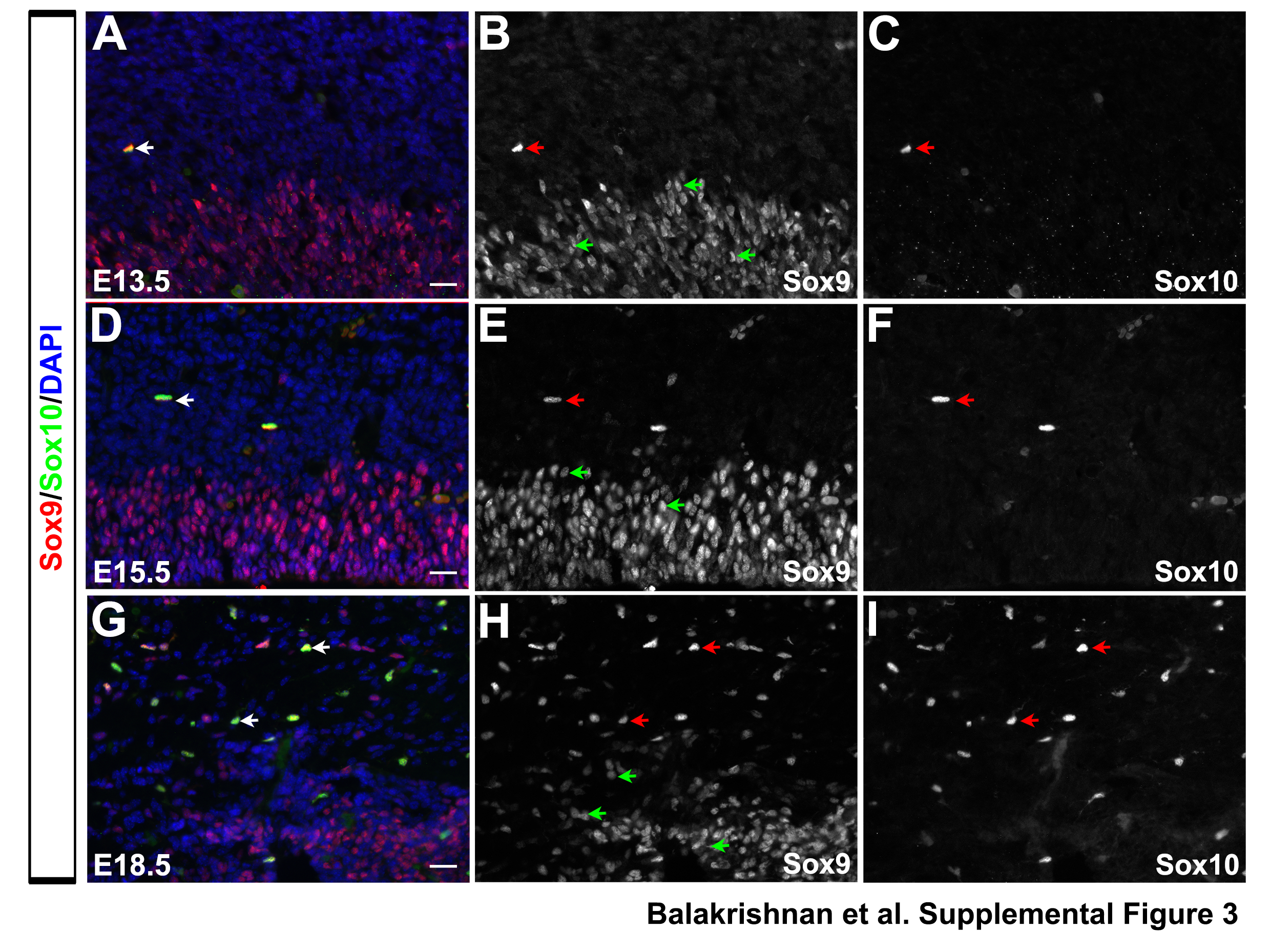

Supplement: S3 Fig — (A-I) Co-labeling of Sox10 with Sox9 in sagittal sections of wild type E13.5 (A-C), E15.5 (D-F), and E18.5 (G-I) cortices. Merged images of Sox9 in red and Sox10 in green (A,D,G). Blue is DAPI counterstain. Expression profiles of Sox9 (B,E,H) and Sox10 (C,F,I). Red arrows indicate co-expression of Sox9 with Sox10, while green arrows mark Sox9+Sox10- cells. Scale bars, 60μm. (TIF) [file pone.0153256.s003.tif]

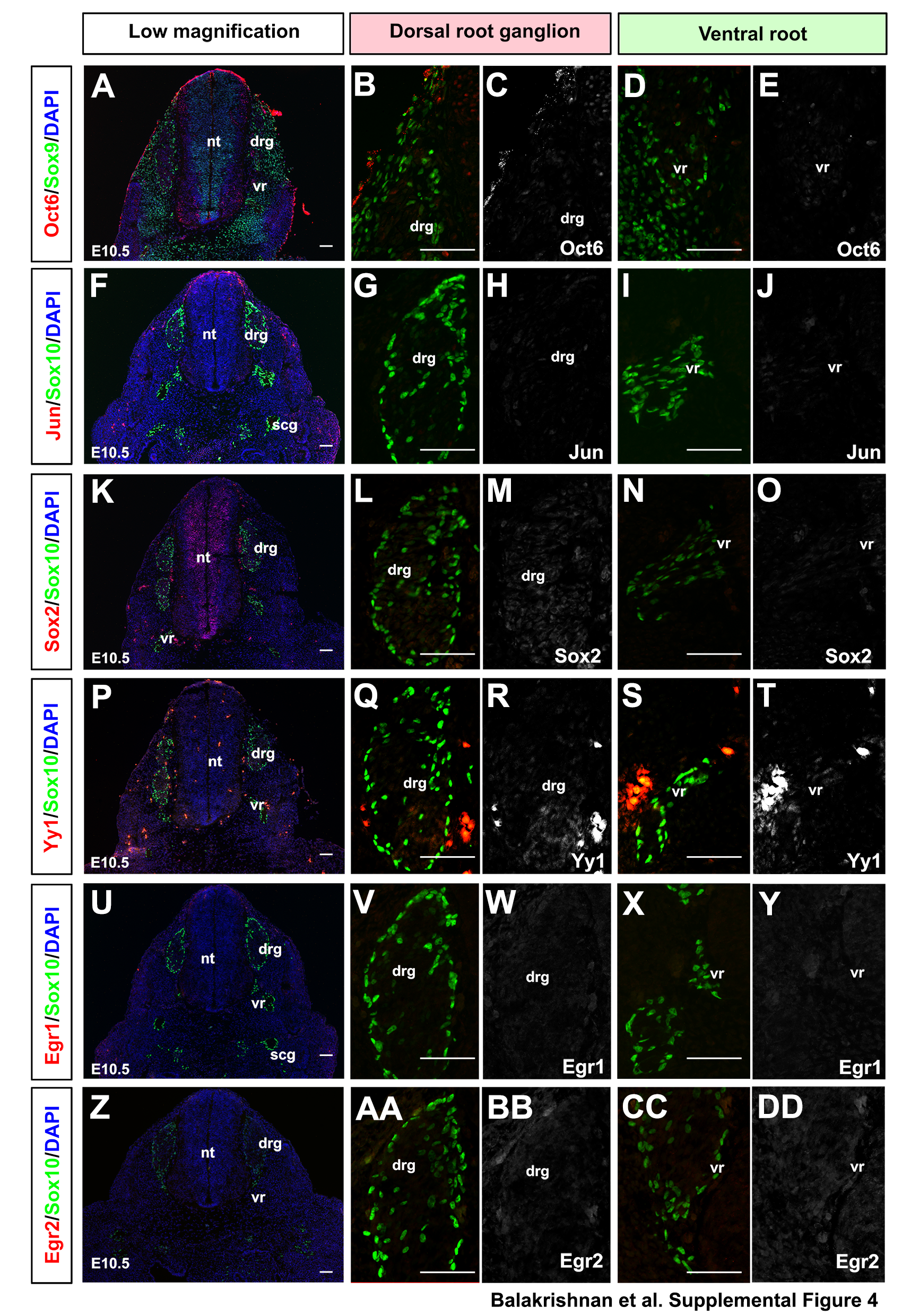

Supplement: S4 Fig — (A-DD) Co-labeling of Sox9 with Oct6 (A-E), and Sox10 with Jun (F-J), Sox2 (K-O), Yy1 (P-T), Egr1 (U-Y), and Egr2 (Z-DD) in transverse sections through the E10.5 trunk. Merged images of the protein of interest (red) with Sox10 or Sox9 (green) (A,F,K,P,U,Z). Blue is DAPI counterstain. High magnification images of the DRG, showing merged images of the protein of interest (red) and Sox10 (green) (B,G,L,Q,V,AA), and single protein of interest images in white (C,H,M,R,W,BB). High magnification images of the ventral roots, showing merged images of the protein of interest (red) and Sox10 (green) (D,I,N,S,X,CC), and single protein of interest images in white (E,J,O,T,Y,DD). drg, dorsal root ganglion; nt, neural tube; scg, sympathetic chain ganglion; sn, spinal nerve; vr, ventral root. Scale bars, 60μm. (TIF) [file pone.0153256.s004.tif]

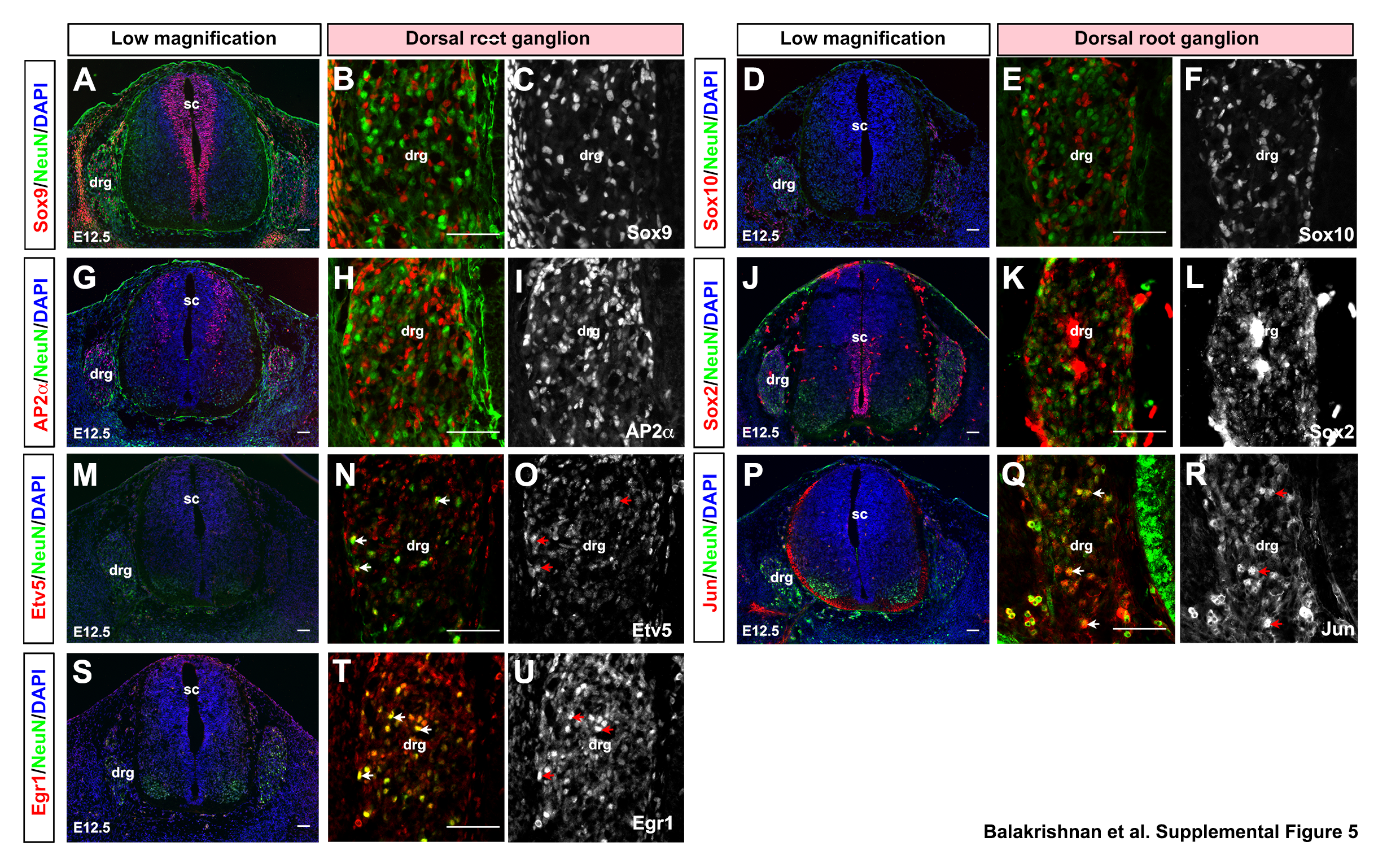

Supplement: S5 Fig — (A-U) Co-labeling of NeuN with Sox9 (A-C), Sox10 (D-F), AP2α (G-I), Sox2 (J-L), Etv5 (M-O), Jun (P-R), Egr1 (S-U). Low magnification merged images of the protein of interest (red) with NeuN (green) (A,D,G,J,M,P,S). Blue is DAPI counterstain. High magnification images of the DRG, showing merged images of the protein of interest (red) and NeuN (green) (B,E,H,K,N,Q,T), and single protein of interest images in white (C,F,I,L,O,R,U). Arrows indicate co-expression of proteins of interest with NeuN in DRG sensory neurons. drg, dorsal root ganglion; sc, spinal cord. Scale bars, 60μm. (TIF) [file pone.0153256.s005.tif]

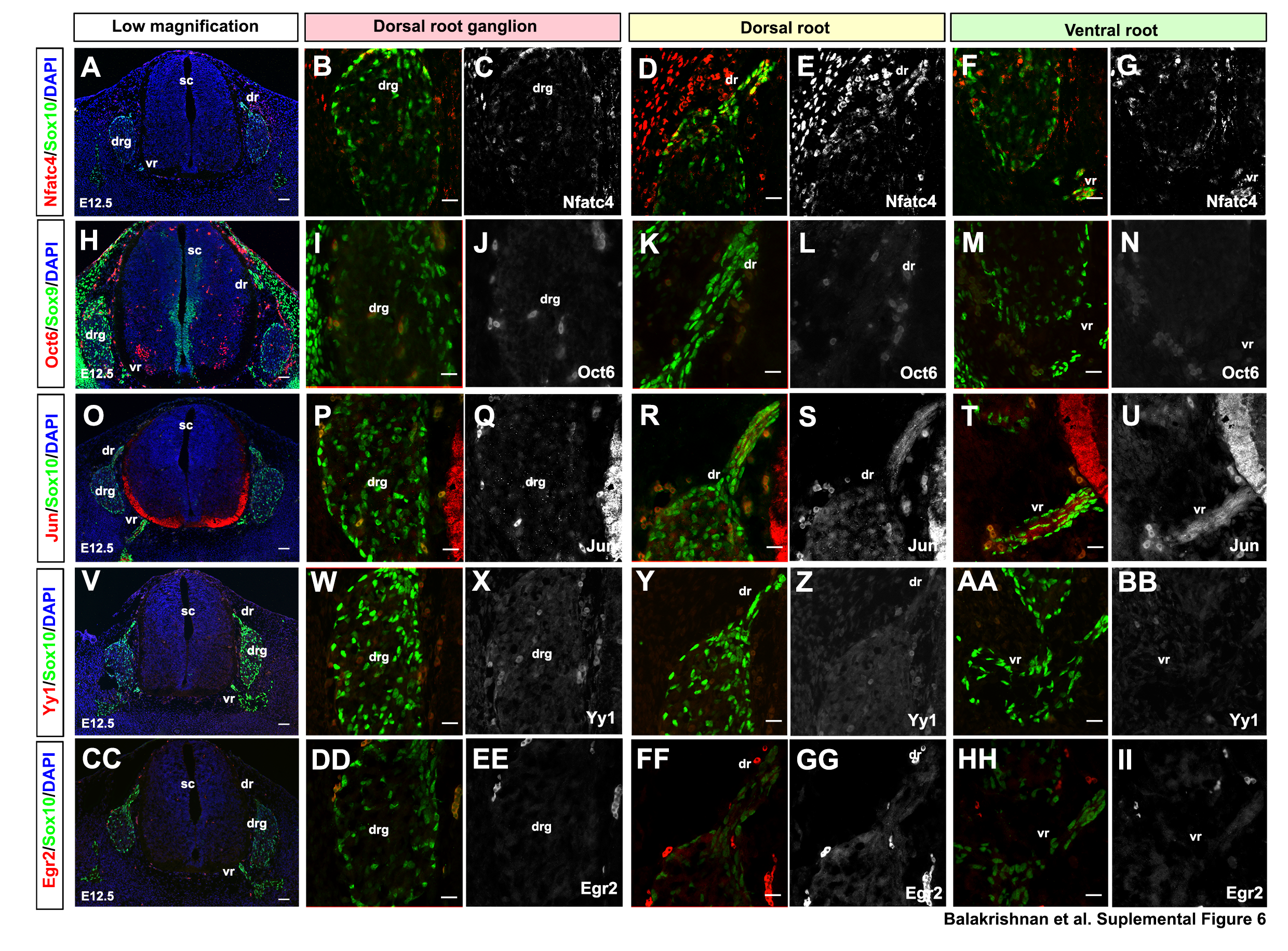

Supplement: S6 Fig — (A-II) Co-expression of Sox9 with Oct6 (H-N), and Sox10 co-expression with Nfatc4 (A-G), Jun (O-U), Yy1 (V-BB), and Egr2 (CC-II) in transverse sections through the E12.5 trunk. Low magnification merged images of protein of interest (red) and Sox9 (green, H) or Sox10 (A,O,V,CC green). Blue is DAPI counterstain. High magnification images of the DRG, showing merged images of the protein of interest (red) and Sox9 (I; green) or Sox10 (B,P,W,DD; green), and single protein of interest images in white (C,J,Q,X,EE). High magnification images of the dorsal roots, showing merged images of the protein of interest (red) and Sox9 (K; green) or Sox10 (D,R,Y,FF; green), and single protein of interest images in white (E,L,S,Z,GG). High magnification images of the ventral roots, showing merged images of the protein of interest (red) and Sox9 (M; green) or Sox10 (F,T,AA,HH; green), and single protein of interest images in white (H,N,U,BB,II). dr, dorsal root; drg, dorsal root ganglion; sc, spinal cord; scg, sympathetic chain ganglion; sn, spinal nerve; vr, ventral root. Scale bars, 60μm. (TIF) [file pone.0153256.s006.tif]

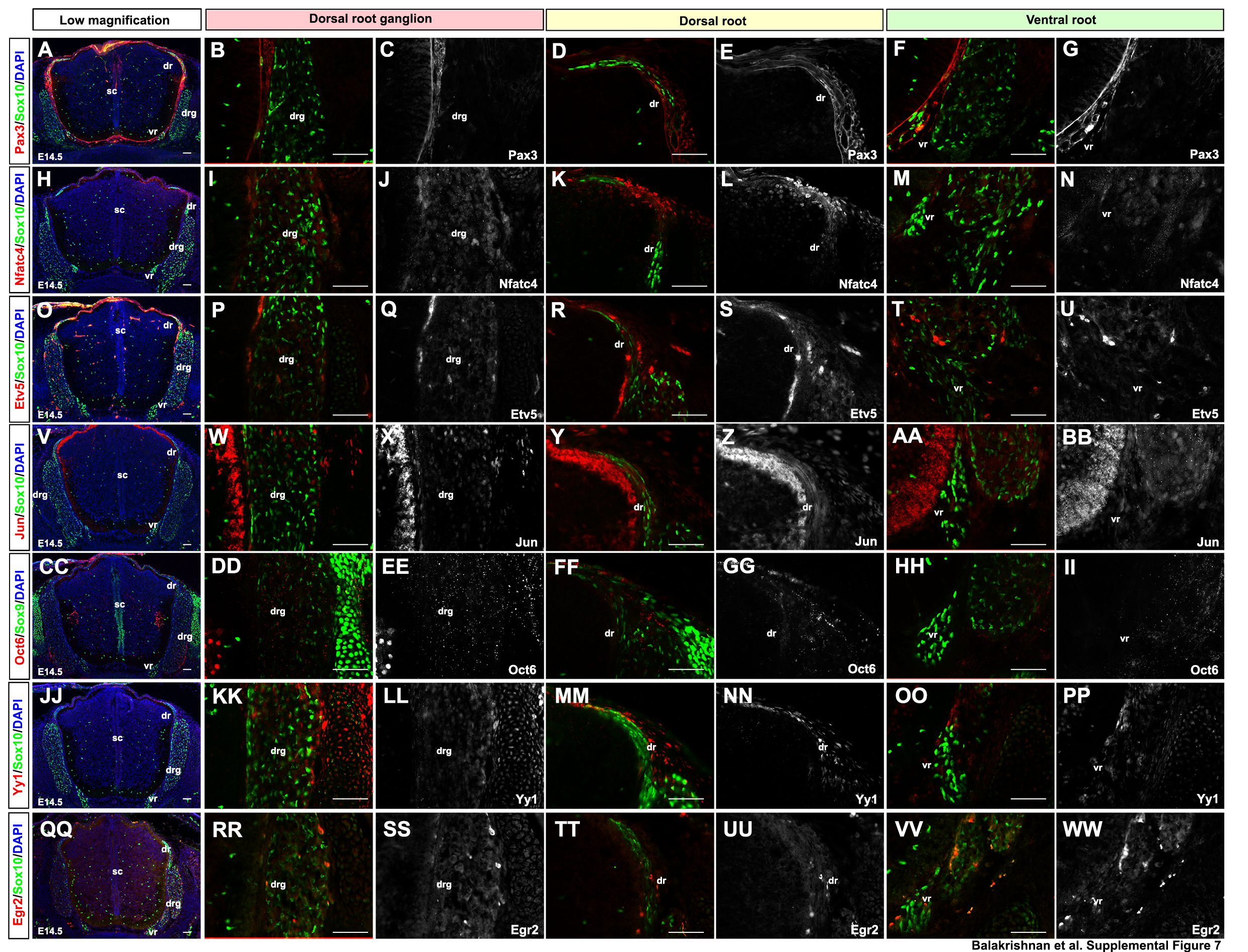

Supplement: S7 Fig — (A-WW) Co-expression of Sox10 with Pax3 (A-G), Nfatc4 (H-N), Etv5 (O-U) Jun (V-BB), Yy1 (JJ-PP), Egr2 (QQ-WW), and Sox9 with Oct6 (CC-II) in transverse sections through the E14.5 trunk. Low magnification merged images of protein of interest (red) and Sox10 (A,H,O,V,JJ,QQ; green) or Sox9 (CC; green). Blue is DAPI counterstain. High magnification images of the DRG, showing merged images of the protein of interest (red) and Sox10 (B,I,P,W,KK,RR; green) or Sox9 (DD; green), and single protein of interest images in white (C,J,Q,X,EE,LL,SS). High magnification images of the dorsal roots, showing merged images of the protein of interest (red) and Sox10 (D,K,R,Y,MM,TT; green) or Sox9 (FF; green), and single protein of interest images in white (E,L,S,Z,GG,NN,UU). High magnification images of the ventral roots, showing merged images of the protein of interest (red) and Sox10 (F,M,T,AA,OO,VV; green) or Sox9 (HH; green), and single protein of interest images in white (G,N,U,BB,II,PP,WW). dr, dorsal root; drg, dorsal root ganglion; sc, spinal cord; vr, ventral root. Scale bars, 60μm. (TIF) [file pone.0153256.s007.tif]

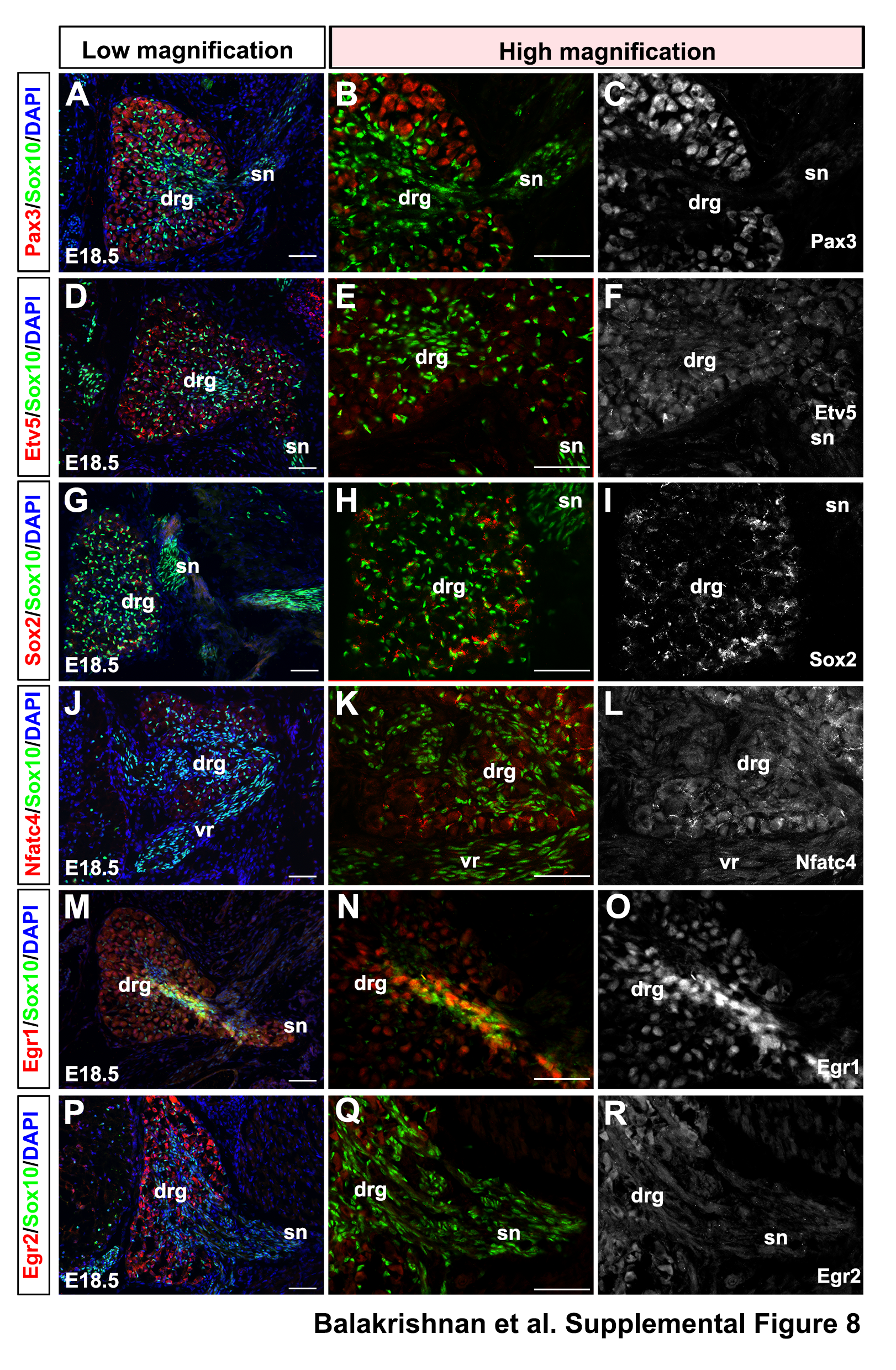

Supplement: S8 Fig — (A-R) Co-labeling of Sox10 with Pax3 (A-C), Etv5 (D-F), Sox2 (G-I), Nfatc4 (J-L), Egr1 (M-O) and Egr2 (P-R). Low magnification merged images of protein of interest (red) and Sox10 (green) (A,D,G,J,M,P). High magnification images of the DRG, showing merged images of the protein of interest (red) and Sox10 (green) (B,E,H,K,N,Q), and single protein of interest images in white (C,F,I,L,O,R). Blue is DAPI counterstain. drg, dorsal root ganglion; sc, spinal cord; sn, spinal nerve; vr, ventral root. Scale bars, 60μm. (TIF) [file pone.0153256.s008.tif]

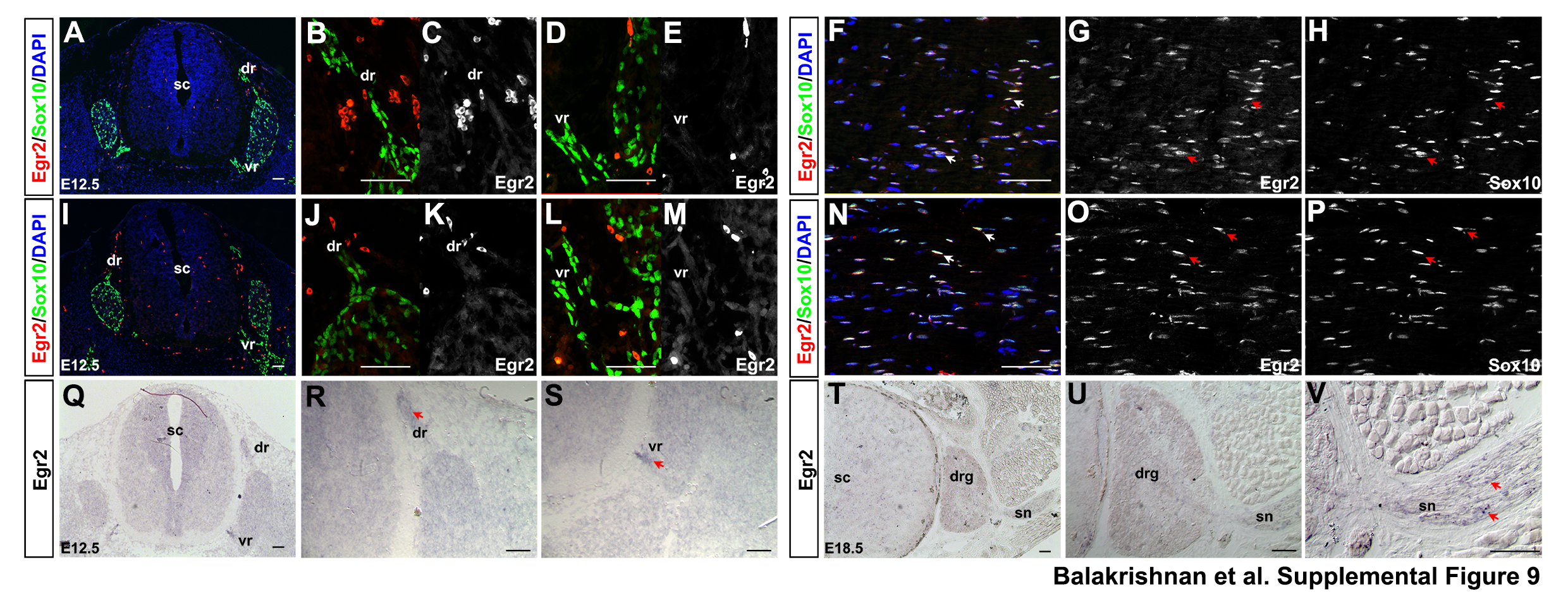

Supplement: S9 Fig — (A-V) Co-labeling of Sox10 with Egr2 (Bioss Antibodies) (A-E & F-H), Egr2 (Abcam) (I-M & N-P) in transverse sections of the E12.5 trunk (A,I) and longitudinal sections of post-natal sciatic nerve (F,N). Merged images of the protein of interest in red and Sox10 in green (A,F,I,N). Blue is DAPI counterstain. RNA in situ hybridisation analysis of Egr2 in transverse trunk sections at E12.5 (Q-S) and E18.5 (T-V). Scale bars, 60μm. (TIF) [file pone.0153256.s009.tif]

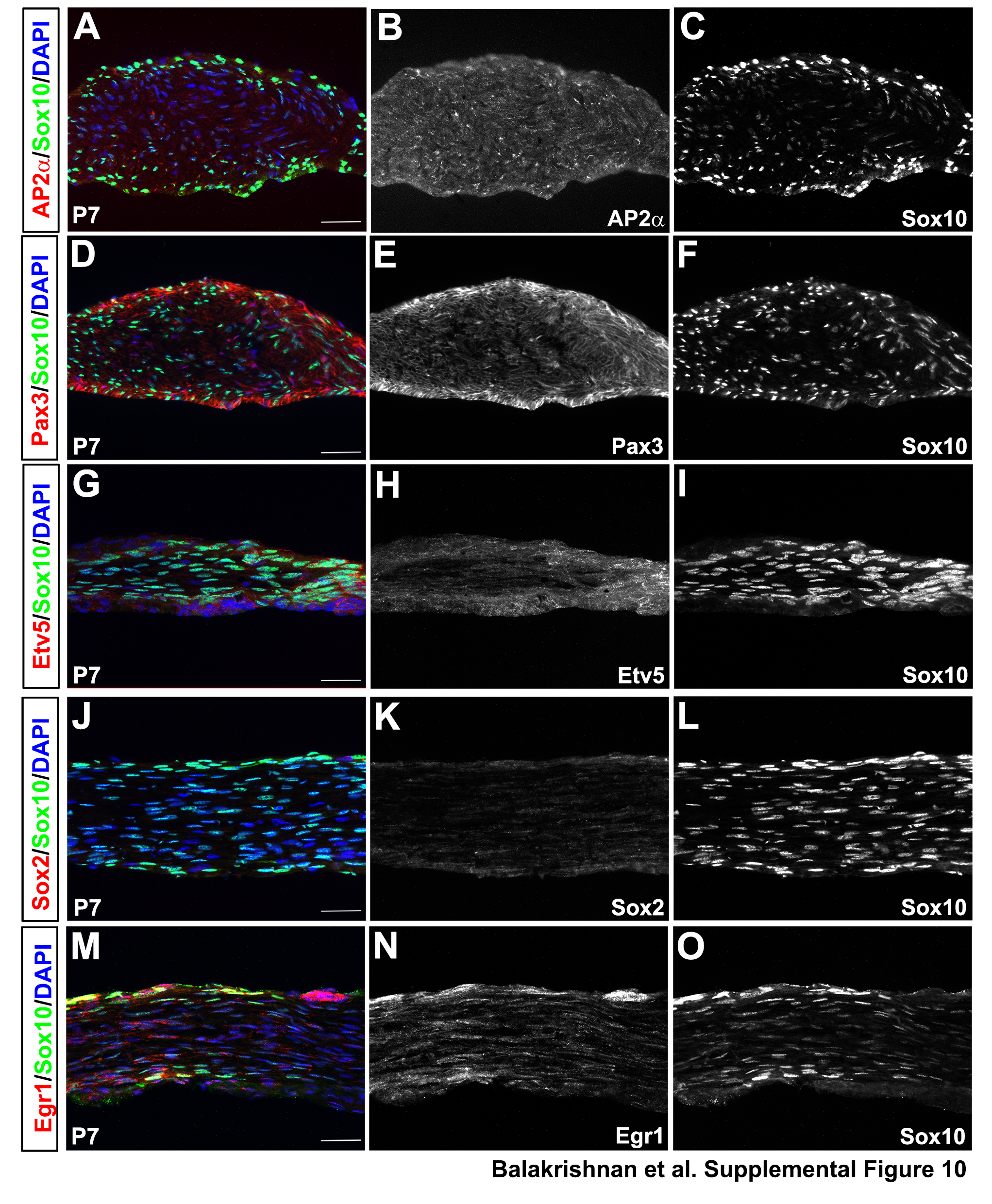

Supplement: S10 Fig — (A-O) Co-labeling of Sox10 with AP2α (A-C), Pax3 (D-F), Etv5 (G-I), Sox2 (J-L), and Egr1 (M-O) in longitudinal sections of the P7 sciatic nerve. Merged images of the protein of interest in red and Sox10 in green (A,D,G,J,M). Blue is DAPI counterstain. Expression profiles of the protein of interest (B,E,H,K,N) and Sox10 (C,F,I,L,O). Scale bars, 40μm. (TIF) [file pone.0153256.s010.tif]

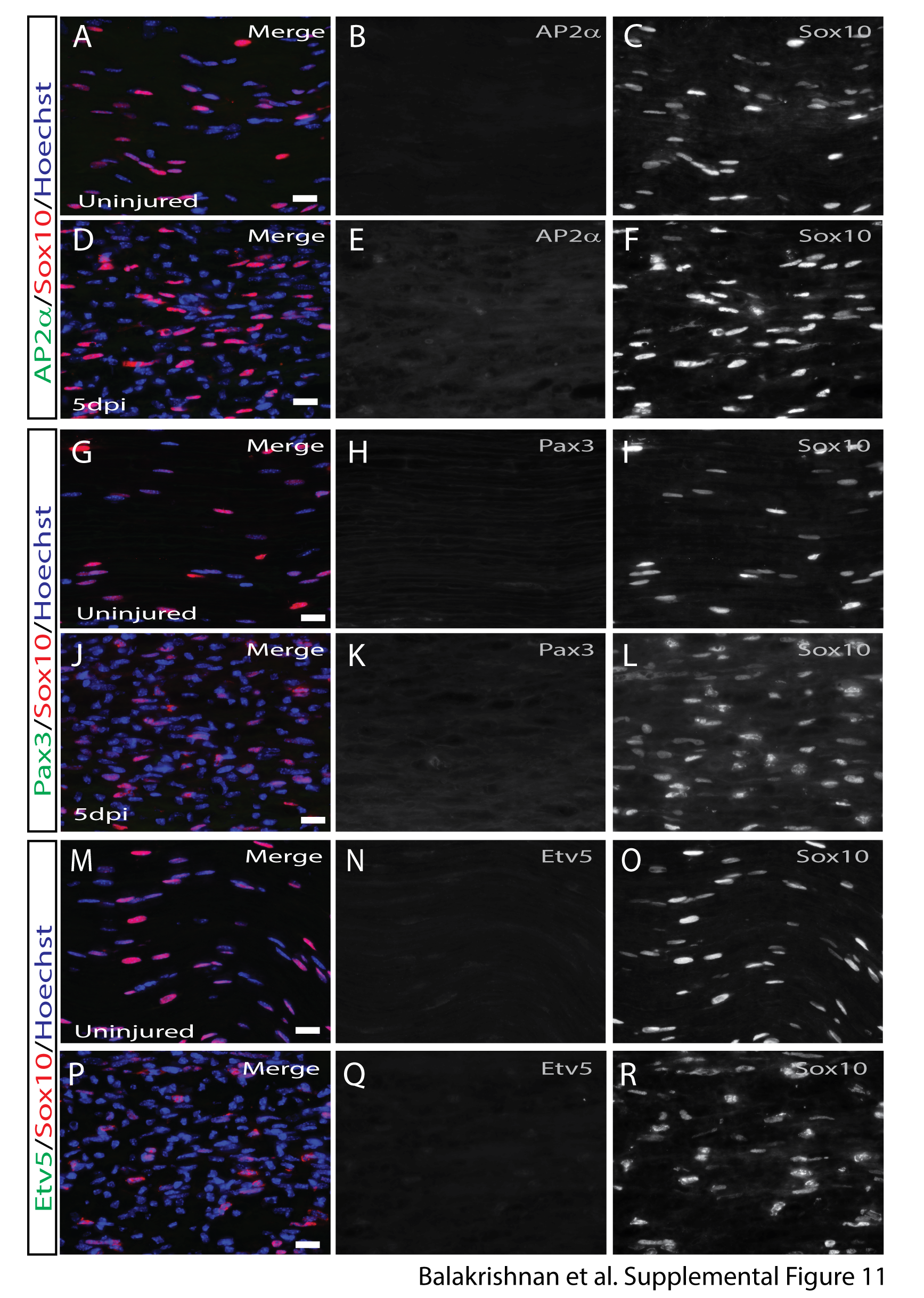

Supplement: S11 Fig — (A-R) Co-labeling of Sox10 with AP2α (A-C & D-F), Pax3 (G-I & J-L), and Etv5 (M-O & P-R). Merged images of protein of interest (green), Sox10 (red) and Hoechst (blue) (A,D,G,J,M,P). Scale bars, 20μm. (TIF) [file pone.0153256.s011.tif]

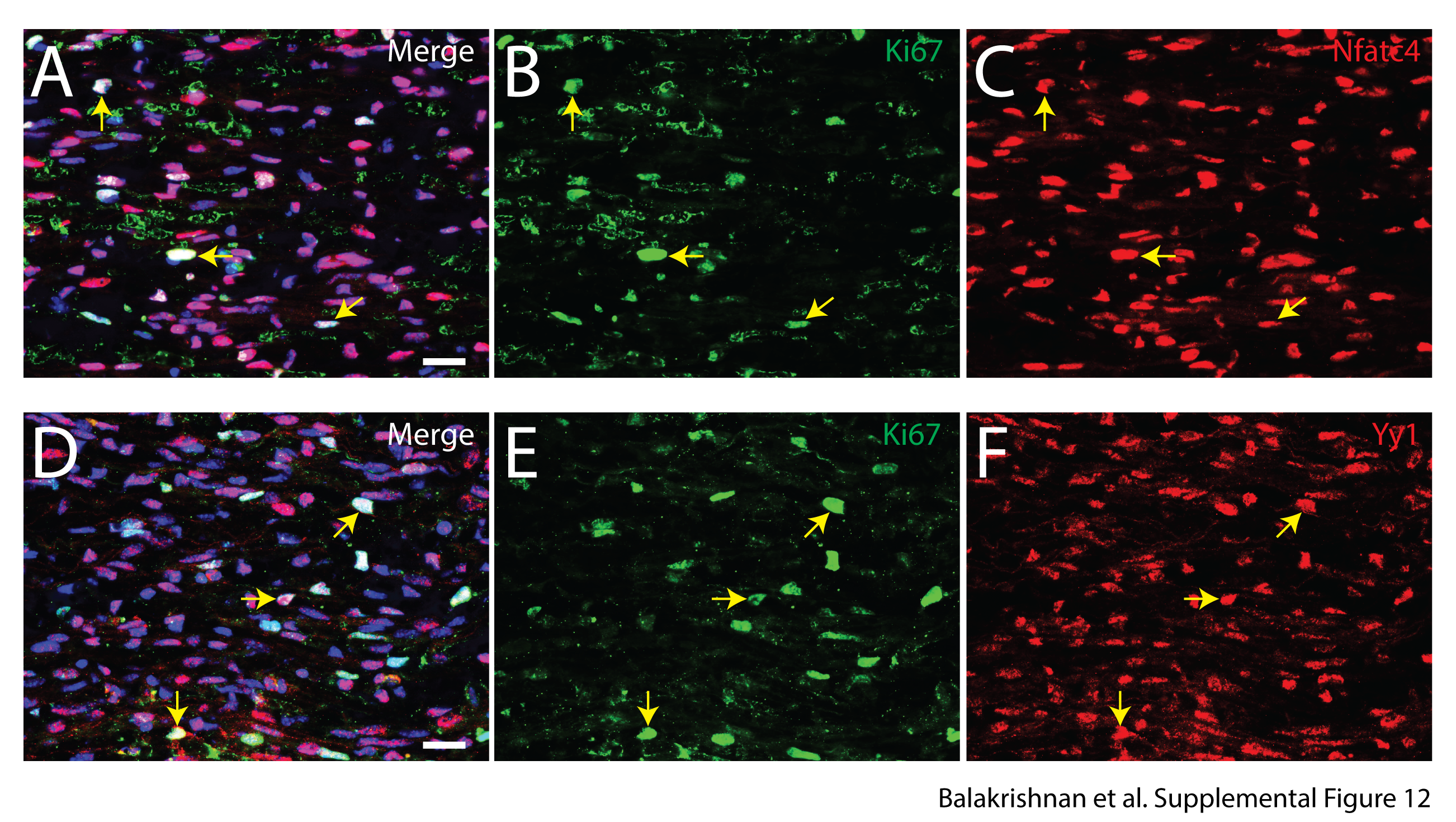

Supplement: S12 Fig — Images showing de-differentiated Schwann cells expressing the promyelinating genes (A-C) Nfatc4 (red) and (D-F) YY1 (red) are mitotically active as indicated by co-localization with Ki67 (green; arrows). Nuclei are stained with Hoechst (blue). Scale bars, 20μm. (TIF) [file pone.0153256.s012.tif]
